# RNA decay during gammaherpesvirus infection reduces RNA polymerase II occupancy of host promoters but spares viral promoters

**DOI:** 10.1371/journal.ppat.1008269

**Published:** 2020-02-07

**Authors:** Ella Hartenian, Sarah Gilbertson, Joel D. Federspiel, Ileana M. Cristea, Britt A. Glaunsinger

**Affiliations:** 1 Department of Molecular and Cell Biology, University of California Berkeley, CA, United States of America; 2 Department of Molecular Biology, Princeton University, Princeton, United States of America; 3 Department of Plant and Microbial Biology, University of California Berkeley, CA, United States of America; 4 Howard Hughes Medical Institute, University of California Berkeley, CA, United States of America; University of North Carolina at Chapel Hill, UNITED STATES

## Abstract

In mammalian cells, widespread acceleration of cytoplasmic mRNA degradation is linked to impaired RNA polymerase II (Pol II) transcription. This mRNA decay-induced transcriptional repression occurs during infection with gammaherpesviruses including Kaposi’s sarcoma-associated herpesvirus (KSHV) and murine gammaherpesvirus 68 (MHV68), which encode an mRNA endonuclease that initiates widespread RNA decay. Here, we show that MHV68-induced mRNA decay leads to a genome-wide reduction of Pol II occupancy at mammalian promoters. This reduced Pol II occupancy is accompanied by down-regulation of multiple Pol II subunits and TFIIB in the nucleus of infected cells, as revealed by mass spectrometry-based global measurements of protein abundance. Viral genes, despite the fact that they require Pol II for transcription, escape transcriptional repression. Protection is not governed by viral promoter sequences; instead, location on the viral genome is both necessary and sufficient to escape the transcriptional repression effects of mRNA decay. We propose a model in which the ability to escape from transcriptional repression is linked to the localization of viral DNA within replication compartments, providing a means for these viruses to counteract decay-induced transcript loss.

## Author summary

While transcription and messenger RNA (mRNA) decay are often considered to be the unlinked beginning and end of gene expression, recent data indicate that alterations to either stage can impact the other. Here we study this connection in the context of lytic gammaherpesvirus infection, which accelerates mRNA degradation through the expression of the viral endonuclease muSOX. We show that RNA polymerase II promoter occupancy is broadly reduced across mammalian promoters in response to infection-induced mRNA decay, and that this phenotype correlates with a reduction in the abundance of several proteins involved in transcription. Notably, gammaherpesviral promoters are resistant to the ensuing transcriptional repression. We show that viral transcriptional escape is conferred by localization of the viral DNA within the protective environment of replication compartments, which are sites of viral genome replication and transcription during infection. Collectively, these findings clarify how mRNA degradation by gammaherpesviruses reshapes the cellular environment and selectively dampens host gene transcription.

## Introduction

Regulating messenger RNA (mRNA) abundance is of central importance during both cellular homeostasis and disease. While it is intuitive that transcriptional changes in the nucleus impact RNA levels in the cytoplasm, evidence in both yeast and mammalian cells now indicates that the converse is also true: altered cytoplasmic mRNA decay rates can broadly impact transcription by RNA polymerase II (Pol II) [1]. In yeast, stabilizing the cytoplasmic mRNA pool by removing mRNA decay factors such as Xrn1 causes a compensatory decrease in Pol II transcription [2–6]. Furthermore, reducing or slowing Pol II transcription leads to an increase in overall mRNA stability [2], supporting a “buffering” model in which yeast can compensate for widespread perturbations to mRNA abundance by alternatively changing rates of decay or transcription.

Broad changes in mRNA abundance are often triggered in mammalian cells during viral infection. Numerous viruses, including alphaherpesviruses, gammaherpesviruses, vaccinia virus, SARS and MERS coronavirus and influenza A virus drive accelerated mRNA decay during infection by expressing mRNA specific ribonucleases and/or by activating host nucleases. This decay contributes to viral immune evasion, increases the availability of host translation machinery, and facilitates temporal viral gene regulation [7–11]. Viral endonucleases target host and viral mRNAs for cleavage, whereupon cellular exonucleases degrade the resulting mRNA fragments [12–16]. This strategy accelerates basal RNA decay by circumventing the typically rate limiting steps of deadenylation and decapping [17–21].

Yeast and mammals share key proteins involved in cytoplasmic mRNA decay and transcription. However, studies with mammalian RNA-decay inducing viruses and viral nucleases have revealed that accelerated mRNA decay is not broadly counteracted by increased transcription-based repopulation, but instead leads to a more extensive shutdown of cellular gene expression. Infection with the gammaherpesviruses KSHV or MHV68 causes reduced Pol II occupancy at individually tested host promoters in a manner dependent on mRNA cleavage by the viral endonucleases SOX or muSOX [22,23]. In uninfected cells expressing muSOX, this Pol II repression phenotype is linked to differential trafficking of RNA binding proteins (RBP) from the cytoplasm to the nucleus [23]. RBP trafficking is thought to occur upon their release from mRNA during the act of transcript degradation, which is initiated by muSOX and completed by cellular exonucleases including Xrn1. It is possible that this altered RBP distribution in cells is interpreted as a stress signal related to pathogenesis, leading to transcriptional dampening rather than the compensatory increase that occurs in other gene regulatory contexts [1].

MHV68 mRNAs are also susceptible to cleavage by muSOX during infection [24]. However, measurements of nascent transcript production from a subset of viral promoters suggested that viral genes are robustly transcribed during the stage of infection when host transcription is reduced [25]. An outstanding question is how these viral promoters escape mRNA decay-induced transcriptional repression despite being transcribed by host Pol II. Here, we measured Pol II occupancy across the mouse and MHV68 genomes in infected cells to more comprehensively define how accelerated mRNA decay impacts polymerase occupancy. Our data demonstrate a genome-wide reduction in Pol II occupancy at host promoters under conditions of increased mRNA decay. However, this phenotype was not reversed upon depletion of cellular exonucleases, suggesting that additional mechanisms contribute to transcriptional repression during infection. Indeed, measurements of global proteome changes by mass spectrometry revealed an mRNA decay-linked decrease in nuclear levels of several transcription factors including key Pol II subunits, which likely contributes to the impact of MHV68 infection on host transcription. Notably, Pol II occupancy of the viral genome appears broadly resistant to the effects of mRNA degradation, despite the change in nuclear levels of Pol II. This protection is not conferred by viral promoter sequences; instead, location on the replicating viral genome is both necessary and sufficient to escape the transcriptional effects of mRNA decay. We propose a model in which DNA amplified in viral replication compartments, unlike the cellular chromatin, is not subject to transcriptional repression during accelerated cytoplasmic mRNA decay. Thus, while both viral and cellular mRNA pools are susceptible to cytoplasmic degradation, transcriptional repopulation selectively counteracts this loss for viral genes.

## Results

### Accelerated RNA decay broadly reduces Pol II occupancy

**Fig 1:**
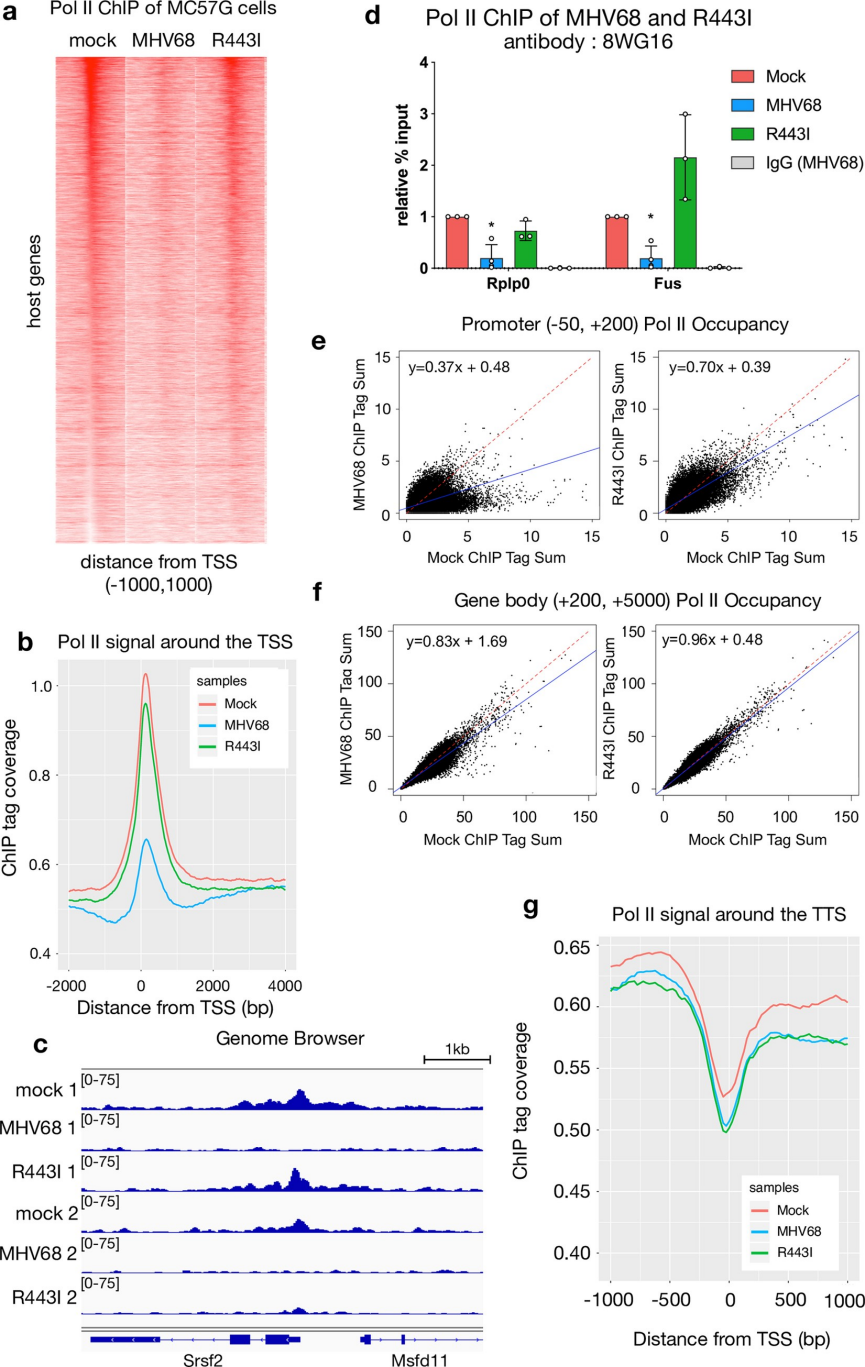
Promoter-proximal Pol II recruitment to mammalian genes is RNA-decay dependent. (A) Pol II ChIP-seq signal profiles of host genes in mock-infected, MHV68 WT-infected and R443I-infected MC57G cells. Each row of the heat map displays Pol II occupancy of one gene from -1000 to +1000 in 25 bp bins. Genes are ranked by the Pol II-transcription start site (TSS) proximal signal in mock infected cells. (B) Sequence tags were plotted as a histogram with 25 bp bins for -2000 to +4000 around the TSS. Mock (red), MHV68 (blue) and MHV68 R443I (green) traces are shown. (C) Pol II ChIP-seq coverage across the *Srsf2* gene for ChIP-seq replicate 1 and 2 with mock, WT MHV68 and R443I shown. Alignment files were converted to the tiled data file (tdf) format and visualized in the Integrative Genome Viewer. (D) ChIP-qPCR validation of Pol II occupancy at the *Rplp0* and *Fus* promoters plotted with standard deviation. Pol II ChIP was performed on mock, MHV68 WT or MHV68 R443I infected MC57G cells and Pol II levels were assayed near the TSS of two repressed host genes during MHV68 infection from the ChIP-seq data. IgG is from the MHV68 infection condition. (* p < 0.05, students paired t-test on raw % input values) (E) Scatterplot of Pol II occupancy of promoters, averaging the sum of ChIP-seq tags from -50 to +200 across two replicate experiments with inputs subtracted out. Mock is compared to WT MHV68 infection on the left and to MHV68 R443I infection on the right. Linear regression lines are plotted in blue and equations are provided. A y = x line is plotted for reference in red. (F) Scatterplot of Pol II occupancy of gene bodies, averaging the sum of ChIP-seq tags from -50 to +200 across two replicate experiments with inputs subtracted out. Data as described for (E). (G) Pol II transcription termination is not dependent on RNA decay. Sequence tags were plotted as a histogram in 25 bp bins for transcription termination sequence (TTS) proximal Pol II for -1000 to +1000 around the TTS with the same color scheme as (B).

We previously reported that Pol II occupancy at several individual mammalian promoters was significantly reduced during accelerated cytoplasmic mRNA decay [23,25]. To more comprehensively assess the extent of mRNA decay-induced transcriptional repression, we evaluated global Pol II occupancy by chromatin immunoprecipitation and deep sequencing (ChIP-seq) in mock versus MHV68 infected MC57G mouse fibroblasts at 24 hours post infection (hpi), which is near the end of the infectious cycle [26]. As a control, we also infected cells with a version of MHV68 containing the point mutation R443I in the viral muSOX endonuclease gene, which reduces its mRNA cleavage activity. MHV68 R443I replicates similarly to WT MHV68 in cultured mouse fibroblasts and in the lungs of infected mice, although it displays *in vivo* defects in viral trafficking to distal sites and latency establishment [7]. MHV68-infected cells showed promoter-proximal loss of Pol II at 86% of loci compared to mock-infected cells, averaged across two biological replicates and subtracting input signal from Pol II occupancy counts from -50 to +200 around the transcription start site (TSS) (Fig 1A and S1A Fig). Loss of Pol II occupancy was primarily due to muSOX-induced mRNA decay, as this signal loss either did not occur or was reduced at 67% of these loci during infection with R443I MHV68 (Fig 1B and S1B Fig). Sixty-three genes, or 1.3% of loci, showed higher Pol II occupancy in WT infection as compared to mock infection, although these did not share any common gene ontology (GO) terms. A representative genome browser view shows reduced Pol II signal throughout *Srsf2* in the MHV68 condition of two replicate ChIP experiments (Fig 1C). The variability between replicates in R443I recovery (Fig 1C) is likely due to differences in the extent of host shutoff reduction between replicates, as R443I reduces muSOX activity but it is not a catalytic mutant. We validated the ChIP-seq results by ChIP-qPCR at the *Rplp0* and *Fus* promoters, both of which showed a significant reduction of Pol II occupancy in cells infected with WT but not R443I MHV68 (Fig 1D). We used a second antibody that recognizes the N-terminus of the major subunit of Pol II, Rpb1, to control for potential bias in the phosphorylation state of the CTD recognized by the ChIP-seq antibody, 8WG16 [27]. We saw a similar decrease in Pol II levels at the promoter with both antibodies as well as a decrease in the *Rplp0* gene body Pol II occupancy with the antibody recognizing the Rpb1 N-terminus upon MHV68 infection (Fig 1D, S1C Fig).

To assess the stage of transcription impacted by RNA decay, we compared the amount of Pol II in promoter regions (-50 to +200) and the amount of Pol II in gene bodies (+200 to +5000) between mock and infected samples. Consistent with the above analyses, MHV68 infected cells contained less promoter proximal Pol II than uninfected cells, and this signal was partially recovered in the R443I infection (Fig 1E). Genes with reduced Pol II promoter binding during MHV68 infection also had less Pol II within the gene body compared to mock infected cells, which again was not observed during R443I infection (Fig 1F). Notably, the difference in regression line slopes between MHV68 and mock infected cells was more marked when comparing Pol II promoter occupancy, suggesting that the primary transcriptional defect is at the stages of recruitment and initiation. This is consistent with data from previous studies that found no defect in serine 2 phosphorylation of elongating Pol II and no consistent change to Pol II elongation during MHV68 infection [23,25].

Finally, to assess if RNA decay affected transcription termination, we plotted sequencing reads between 1kb upstream and 1kb downstream of the transcription termination site (TTS) as a histogram (Fig 1G and S1D Fig). TTS-proximal Pol II shows no defect in termination in either infection condition, suggesting Pol II removal from host transcripts is not impacted by MHV68 infection or by RNA decay.

### Depletion of host RNA decay factors is not sufficient to abrogate feedback between mRNA decay and Pol II occupancy

**Fig 2:**
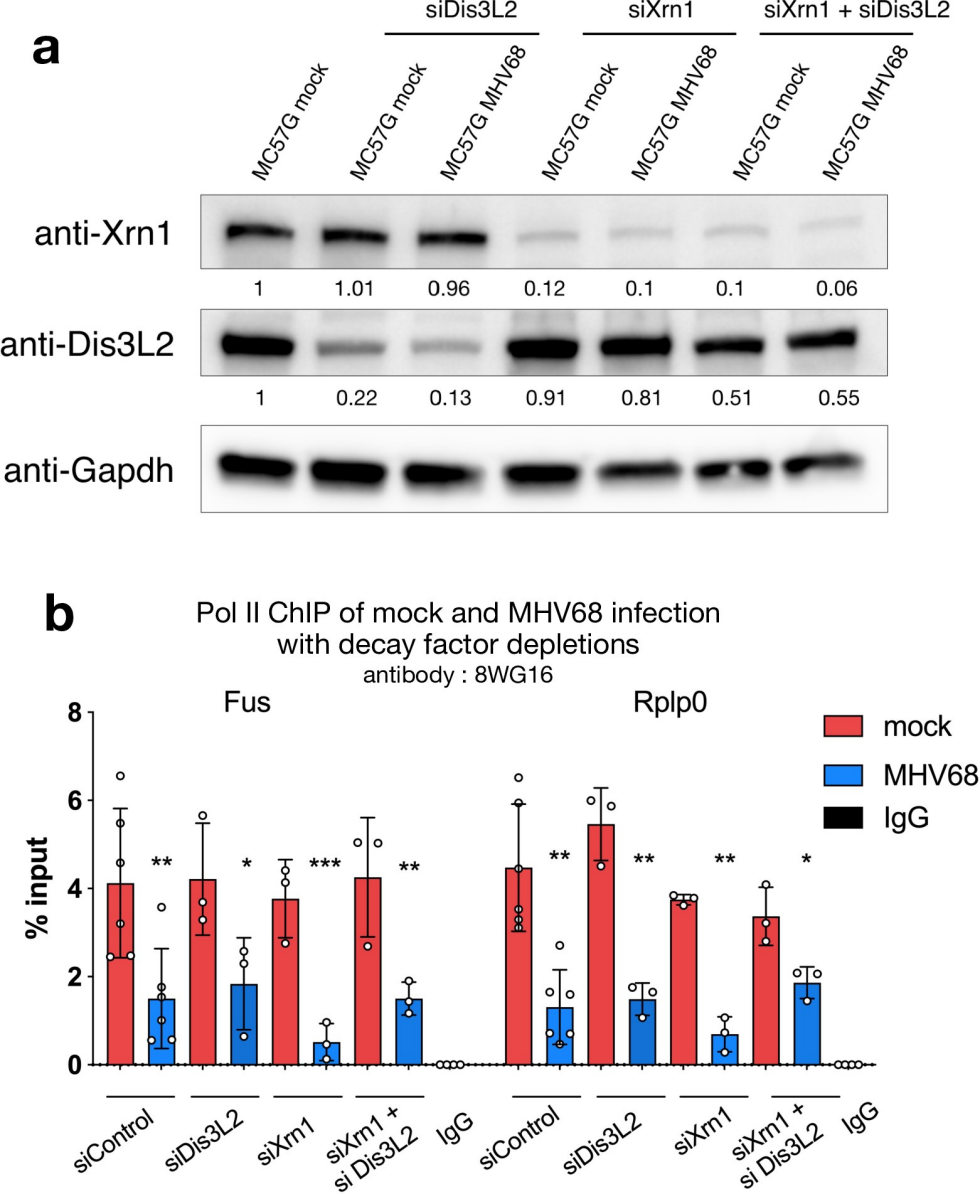
Knockdown of host RNA decay factors does not rescue Pol II occupancy. (A) Representative western blot showing the efficiency of Dis3L2 and Xrn1 knockdown (kd) in MC57G cells, with Gapdh serving as a loading control. Densitometry of band intensity normalized to Gapdh and MC57G mock infection are indicated below blots. (B) ChIP-qPCR of Pol II occupancy at the *Rplp0* and *Fus* promoters with standard deviation. Pol II ChIP was performed on mock and MHV68 WT infected MC57G cells treated with the indicated siRNAs. IgG is from the siControl MHV68 infection condition. (* p < 0.05, ** p < 0.001, *** p < 0.0001 students paired t-test on raw % input values).

muSOX expression alone is sufficient to reduce cytoplasmic populations of mRNA by 60–80% [28–30], and in this non-infectious setting the loss of promoter-proximal Pol II is dependent on the presence of the host RNA decay enzymes Xrn1 and Dis3L2 [25]. To determine if this mechanism is also sufficient to explain the loss of Pol II occupancy during MHV68 infection, we depleted Xrn1 and Dis3L2 individually or in tandem using siRNAs (Fig 2A). Unexpectedly, depletion of these cellular decay factors did not reproducibly rescue Pol II occupancy at the *Fus* and *Rplp0* promoters during MHV68 infection compared to mock infected cells (Fig 2B). These data suggest that additional factors contribute to the mRNA decay-dependent decrease in Pol II from cellular promoters during MHV68 infection.

### General transcription factors and Pol II subunits are depleted in the nucleus in an RNA decay dependent manner

**Fig 3:**
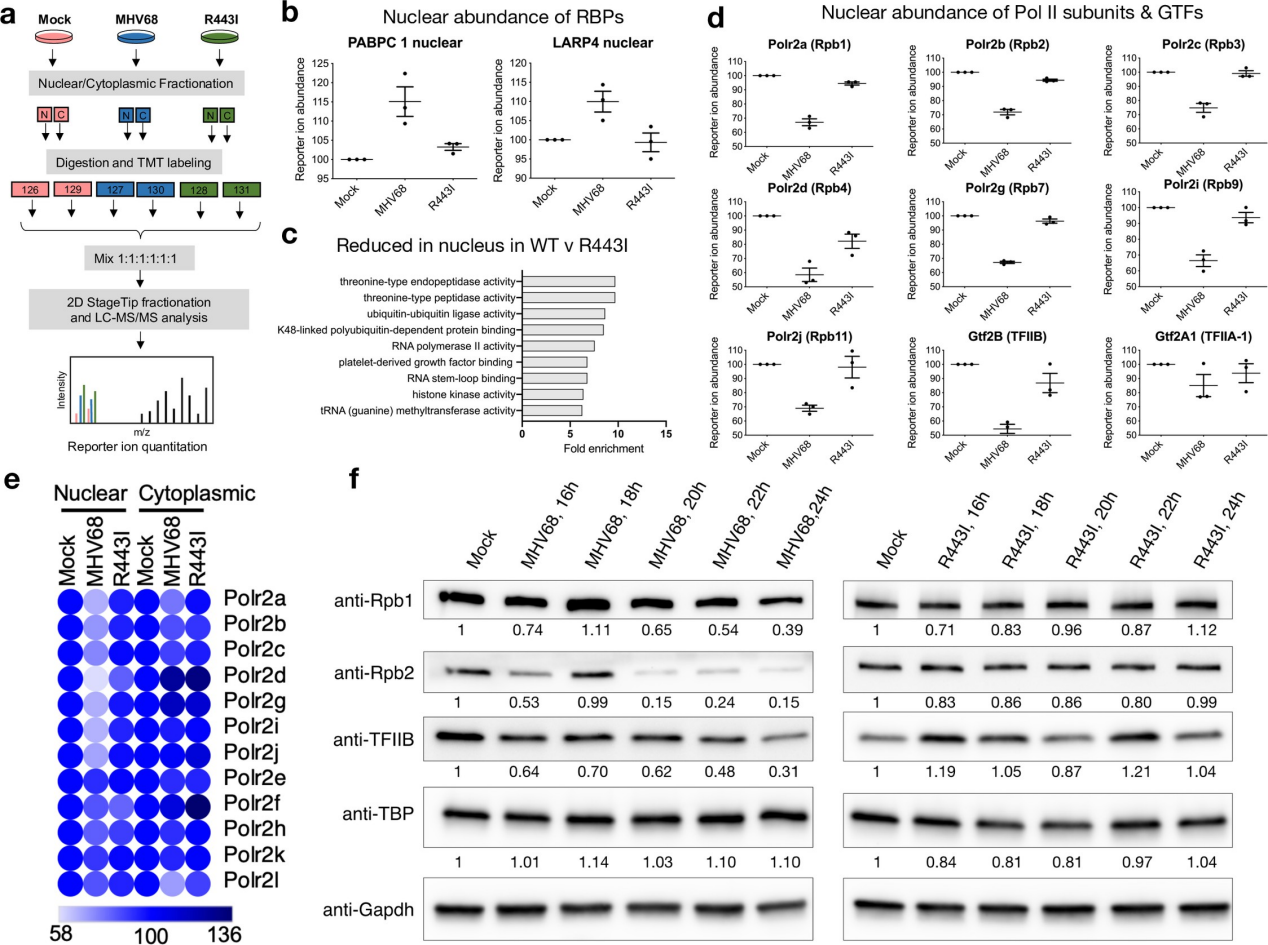
Pol II subunits are depleted in the nucleus of MHV68 infected cells in an RNA decay dependent manner. (A) Diagram showing the experimental setup for the TMT-MS. (B) Graphs showing the nuclear distribution of poly(A) binding proteins from the TMT-MS data. Graphs display the mean with SEM of 3 biological replicates. (C) Molecular functions classification by Pantherdb of nuclear proteins differentially expressed by 1.15 fold or more in R443I compared to WT MHV68 infection of 3T3 cells in the TMT-MS data. Categories with fold enrichment greater than 5 are shown. (D) Graphs showing the relative nuclear abundance of 7 Pol II subunits and 2 general transcription factors from the TMT-MS data that are reduced in MHV68 infection compared to R443I. Graphs display the mean with SEM of 3 biological replicates. (E) A heatmap displaying normalized reporter ion abundance of all 12 Pol II subunits in the nucleus and cytoplasm, where light blue represents lower abundance and dark blue represents higher abundance. (F) Representative western blot showing the levels of Rpb1, Rpb2, TFIIB and TBP upon mock infection versus infection with WT MHV68 or R443I MHV68 in MC57G cells at the indicated times post infection in whole cell lysate. Gapdh serves as a loading control. Densitometry of band intensity was normalized first to Gapdh and then mock infection are indicated below blots.

To identify other proteins that might contribute to the reduced Pol II occupancy at host promoters during infection, we applied an unbiased mass spectrometry-based approach to measure protein abundance and localization differences in 3T3 mouse fibroblast cells that were mock infected or infected with WT or R443I MHV68 at 24 hpi. We previously showed that accelerated mRNA decay by the coordinated activity of muSOX and Xrn1 causes RNA binding protein relocalization from the cytoplasm to the nucleus and that this relocalization plays a role in transcriptional repression in 293T cells [23]. We therefore performed a similar experiment in infected cells, in which each sample was separated into nuclear and cytoplasmic fractions and proteins from each fraction were labeled with isobaric tandem mass tags (TMT). We then multiplexed these TMT labeled fractions and subjected them to quantitative liquid chromatography/tandem mass spectrometry (LC/MS-MS) (Figs 3A and S2A, S1 Table). In agreement with our previous findings [23], we observed the expected increase in nuclear levels of the RNA binding proteins PABPC1 and LARP4 in cells infected with WT MHV68 compared to mock or R443I infected cells (Fig 3B).

We then searched for additional proteomic differences specific to WT MHV68 infected cells that might contribute to decreased Pol II occupancy. We examined classes of proteins that increased or decreased in a host shutoff dependent manner in the nucleus and the cytoplasm (S2 B-D). Notably, RNA Polymerase II activity-associated proteins were substantially decreased in WT MHV68 infection compared to R443I or mock cells (Fig 3C). The nucleus-specific changes included decreased levels of 7 subunits of Pol II, the general transcription factor (GTF) TFIIB, and the large subunit of the GTF TFIIA (Fig 3D). The lack of a corresponding increase in the cytoplasmic levels of Pol II subunits suggests that muSOX activity decreased their overall abundance rather than changing their localization (Figs 3E and S2D). We confirmed this hypothesis by measuring the abundance of several of these proteins by western blotting whole cell lysates across a time course of WT and R443I MHV68 infection in MC57G cells (Fig 3F). WT MHV68 infection reduced the levels of Pol II subunits Polr2a (Rpb1), Polr2b (Rpb2) and Gtf2B (TFIIB) beginning at 20 hpi. In contrast, levels of the GTF TATA Binding Protein (TBP) are not decreased in either the TMT dataset or in the western blotting experiments (Fig 3F, S1 Table). R443I virus infection showed relatively constant levels of Rpb1, Rpb2, TFIIB and TBP across the time course (Fig 3F). Collectively, these findings demonstrate that muSOX-induced mRNA degradation during MHV68 infection causes both RNA binding protein relocalization and depletion of multiple components of the cellular transcriptional machinery. It is likely that this combination of phenotypes contributes to the broad reduction in Pol II occupancy at host promoters.

### Pol II occupancy at viral genes is not affected by RNA decay

**Fig 4:**
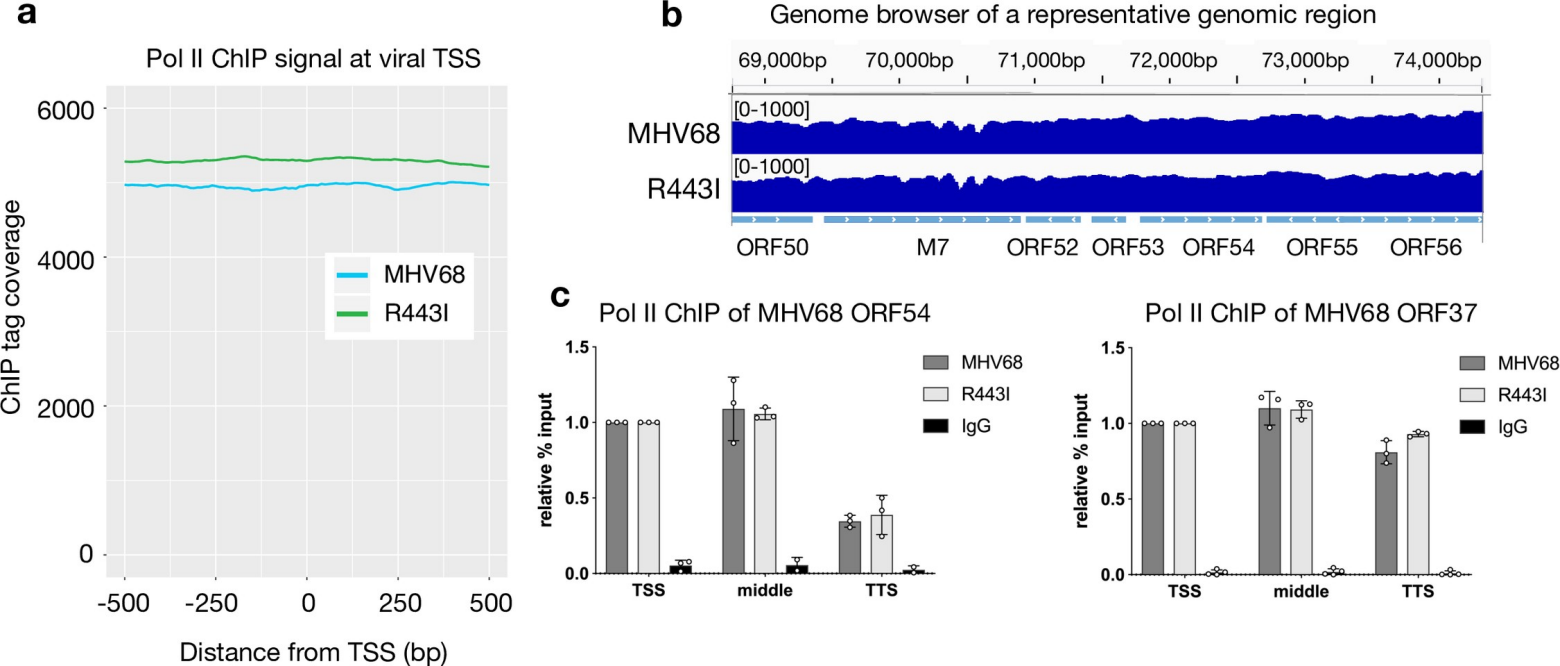
Viral genes are not susceptible to RNA decay-dependent Pol II repression. (A) Sequence tags were plotted as a histogram in 25 bp bins for -500 to +500 around the TSS. MHV68 WT (blue) and R443I (green) traces are shown from infected MC57G cells. (B) Pol II ChIP-seq coverage across 5.5 kb of the MHV68 genome for MHV68 WT and R443I. Alignment files were converted to tdf format and visualized in the IGV. (C) Pol II ChIP qPCR signal is similar across gene bodies. Three regions of the ORF54 and ORF37 gene were assayed by Pol II ChIP-qPCR during MHV68 WT or R443I infection to assess the relative levels of Pol II across the gene.

Herpesviral genes are transcribed in the nucleus by mammalian Pol II, and thus could be similarly subjected to the mRNA decay-induced transcriptional repression observed across the host genome. We therefore analyzed Pol II occupancy of the viral genome in WT MHV68 and R443I infected cells to determine how viral genes respond to RNA decay in light of the decrease in Pol II subunits. The average Pol II signature across 82 viral ORFs between -500 and +500 around the transcription start site was comparable in cells infected with WT or R443I MHV68, with a slightly higher signal in the R443I infection (Fig 4A). Indeed, as shown in the genome browser of a representative genomic region, there was a robust and widespread Pol II signal in both infection conditions (Fig 4B). To determine if transcription from the viral genome was affected by host shutoff, we pulse labeled WT MHV68 and R443I infected cells with 4-thiouridine (4SU) for 10 min to measure nascent transcript levels. 4SU gets incorporated into actively transcribing mRNAs and can be subsequently coupled to MTSEA -biotin and purified over streptavidin beads [59]. For the 7 transcripts we measured, we saw no statistically significant difference in nascent transcription comparing WT to R443I (S3 Fig), consistent with our Pol II ChIP-seq results. Thus, herpesviral genes appear to escape mRNA decay-induced transcriptional repression.

In contrast to host genes, we did not observe peaks of promoter-proximal Pol II on the viral genome in our ChIP-seq data (Fig 4A and 4B). Pol II ChIP qPCR across various regions of ORF54 and ORF37 also showed a similar Pol II signal throughout each gene body and at their promoter. We did see a decrease in signal near the TTS of ORF54, however this was true for both WT and R443I infections (Fig 4C). We hypothesize that pervasive transcription of the viral genome at 24 hpi, compounded by the presence of TSSs on both Watson and Crick strands, likely contributes to this broad Pol II signal.

### PABPC is not excluded from viral replication compartments

**Fig 5:**
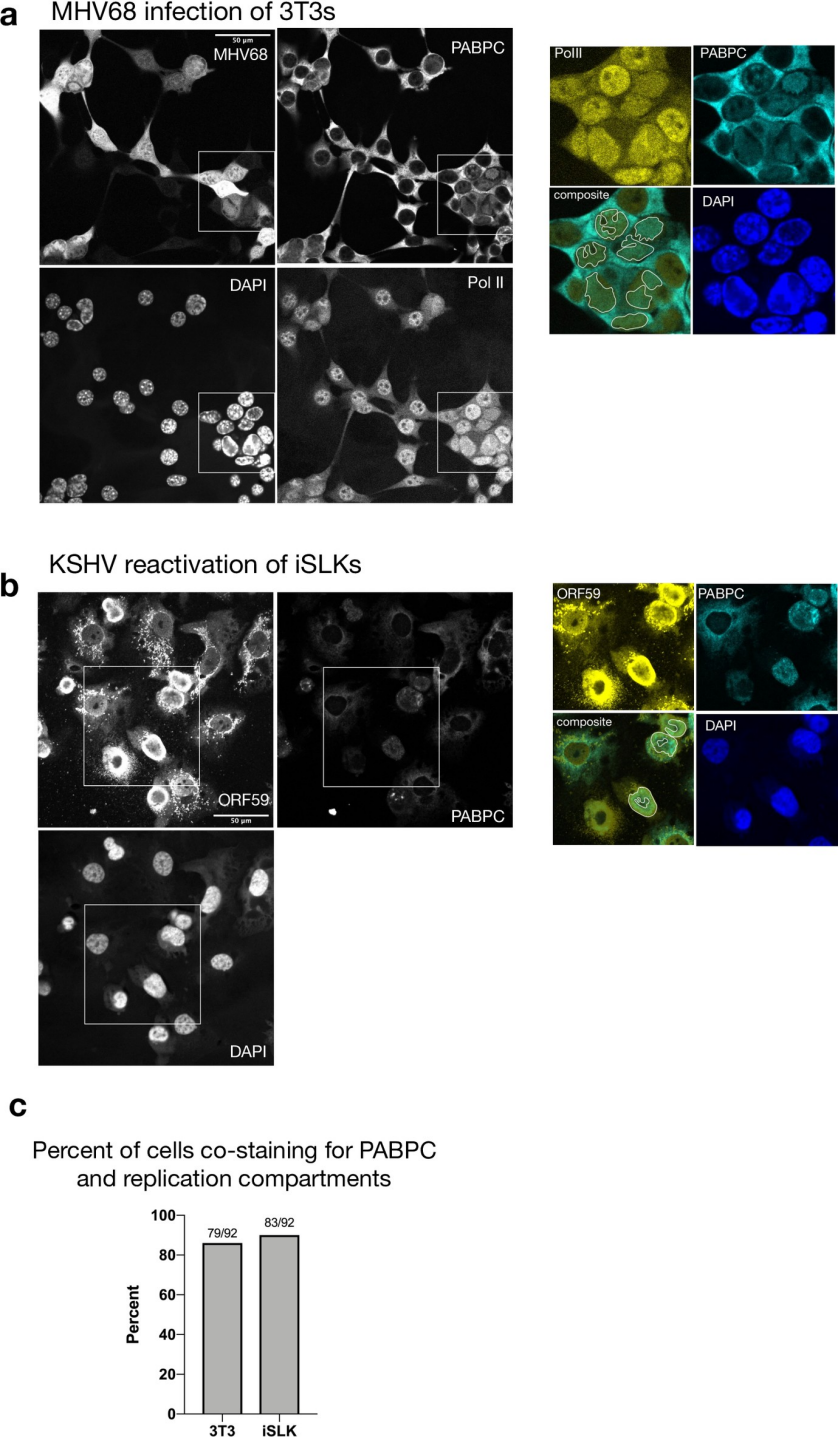
PABPC is not excluded from replication compartments. (A) MHV68 infected 3T3 cells were subjected to imunofluoresence (IF) analysis at 27 hpi using antibodies against PABPC and Pol II, and stained with DAPI. The MHV68 genome contains GFP, which served as a marker of infection. Cells with RCs are outlined in white. RCs were identified in cell nuclei as regions that contained Pol II but stained poorly for DAPI. The inset shows a merge of Pol II and PABPC staining for several cells that co-stain for both proteins in RCs. (B) IF was performed on KSHV-positive iSLK cells reactivated for 48 h and stained with antibodies against PABPC, ORF59 and DAPI. Cells with RCs are outlined in white and were identified as nuclei with ORF59 staining that overlaps with regions that stain poorly with DAPI. The inset shows a merge of ORF59 and PABPC for several cells that co-stain for both proteins in RCs. (C) Percent of RC containing cells that overlap with PABPC signal. Fractions of cells with PABPC signal in RCs over total number of cells counted with RCs are displayed.

We next considered how the viral genome maintains high levels of Pol II occupancy during accelerated mRNA decay. During replication, viral DNA is transcribed within viral replication compartments (RCs) in the nucleus, which exclude host DNA and thus stain poorly with DAPI [31–33]. Although they are not membrane bound, these compartments selectively enrich for factors required for viral replication (such as the viral processivity factor ORF59) and gene expression (such as Pol II) [34–36]. Virus-induced mRNA decay causes increased trafficking of cytoplasmic poly(A) binding protein (PABPC) from the cytoplasm to the nucleus (see Fig 3B), which has been linked to transcriptional repression [23,37]. To determine whether replication compartments selectively exclude nuclear PABPC as a means of avoiding transcriptional repression, we monitored its localization in cells lytically infected with MHV68 or KSHV. As expected, the majority of infected cells displayed increased nuclear PABPC compared to unreactivated or uninfected cells as measured by immunofluorescence (IF). However, PABPC did not appear to be excluded from replication compartments (Fig 5A and 5B and S4 Fig). To confirm PABPC localization relative to RCs, we first identified cells with RCs by looking for nuclear regions containing Pol II or ORF59 that also showed reduced DAPI staining. We then quantified nuclear PABPC pixel intensity, and counted cells as having an overlapping RC/PABPC signal when the PABPC pixel intensity was at least 2x that of cells without nuclear PABPC from the same image. Indeed, 85 percent of MHV68 infected cells showed overlapping PABPC/Pol II signal and 90 percent of reactivated iSLKs showed an overlapping PABPC/ORF59 signal (Fig 5C). Thus, PABPC exclusion from RCs is unlikely to underlie viral promoter escape.

### Viral promoters fail to escape transcriptional repression outside of the viral genome

**Fig 6:**
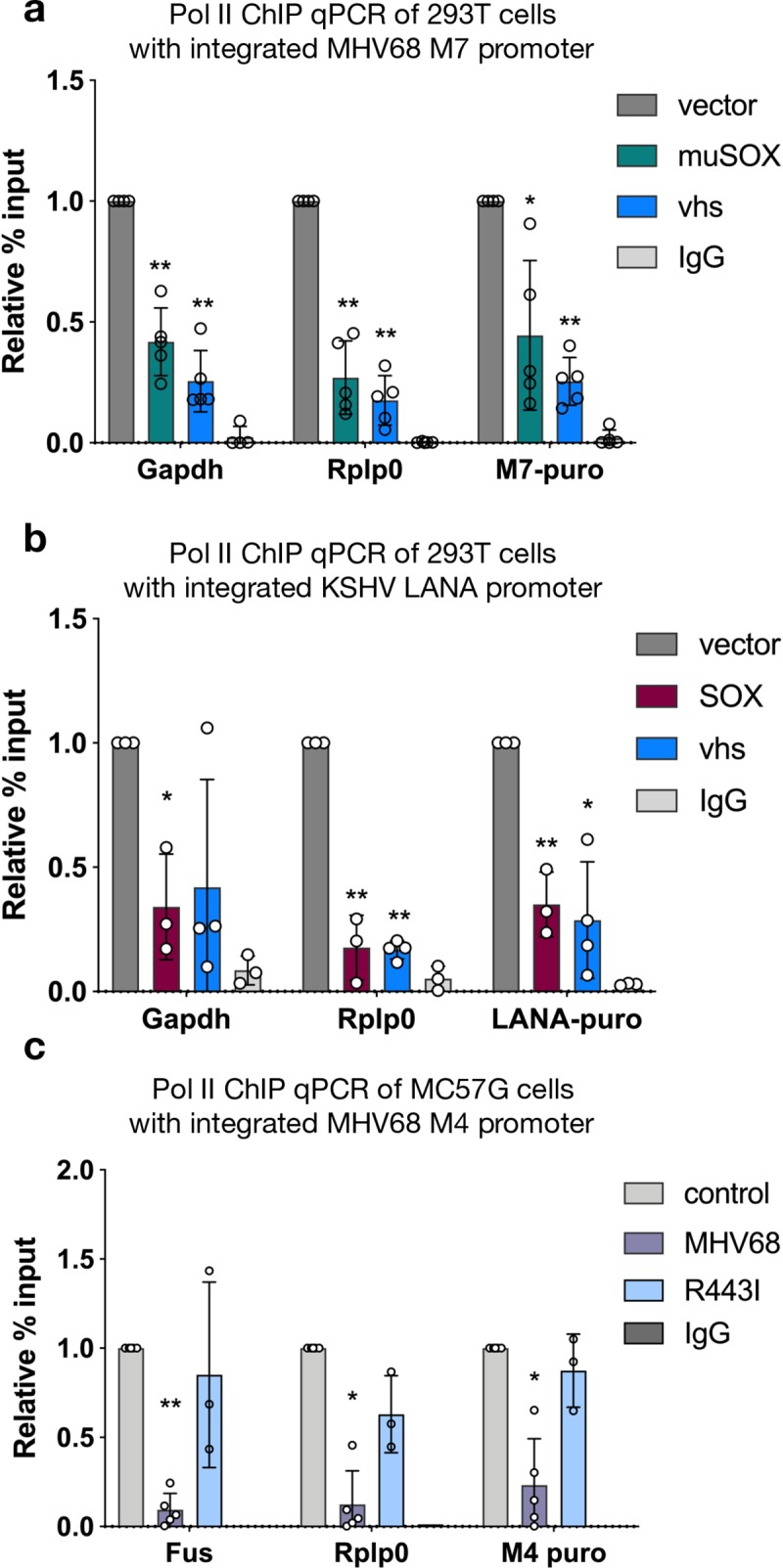
Viral promoter sequences are insufficient to escape transcriptional regulation. (A) The MHV68 M7 viral promoter driving puromycin resistance was lentivirally integrated into 293T cells. 24 h post transfection of muSOX or vhs, Pol II-ChIP qPCR was used to measure Pol II levels at two host promoters (*Gapdh*, *Rplp0*) and the integrated M7 promoter. (* p < 0.05, ** p < 0.001, students paired t-test on raw % input values plotted with standard deviation) (B) The KSHV LANA promoter driving puromycin resistance was integrated into 293T cells by random incorporation. Pol II-ChIP qPCR was used to measure Pol II levels 24 h post SOX or vhs transfection as described in (A) and significance as assigned in (A). (C) The MHV68 M4 viral promoter driving puromycin resistance was lentivirally integrated into MC57G cells. The cells were then infected with WT MHV68 or R443I at a MOI of 5 and Pol II levels at the indicated promoters were assayed by Pol II ChIP-qPCR. Significance as assigned in (A).

We next tested whether viral promoter sequence elements were sufficient to enable escape by querying whether they conferred resistance to transcriptional repression upon relocation from the viral genome into the host DNA. We inserted the MHV68 late promoter M7 or the KSHV LANA promoter upstream of a puromycin resistance cassette into 293T cell chromatin using either lentiviral transduction or random plasmid DNA integration, respectively. We induced mRNA decay and host transcriptional repression in these cells by expression of MHV68 muSOX or the herpes simplex virus type 1 (HSV-1) endonuclease vhs. Similar to the host promoters *Gapdh* and *Rplp0*, Pol II occupancy of both integrated viral promoters was repressed upon transfection with muSOX and vhs (Fig 6A and 6B).

To confirm that viral promoters integrated into the host genome were also repressed in the context of infection, we infected MC57G cells containing an integrated MHV68 M4 promoter-driven puromycin cassette with MHV68. Indeed, the M4 promoter was repressed to a similar degree as host *Fus* and *Rplp0* promoters during infection with WT MHV68 but not the mRNA decay-deficient MHV68 R443I at 24hpi (Fig 6C). In summary, these data argue against the presence of protective elements within viral promoters, and instead suggest that the chromatin context or location on the viral genome underlies escape from mRNA decay-induced transcriptional repression.

### A non-MHV68 promoter escapes repression on the viral genome

**Fig 7:**
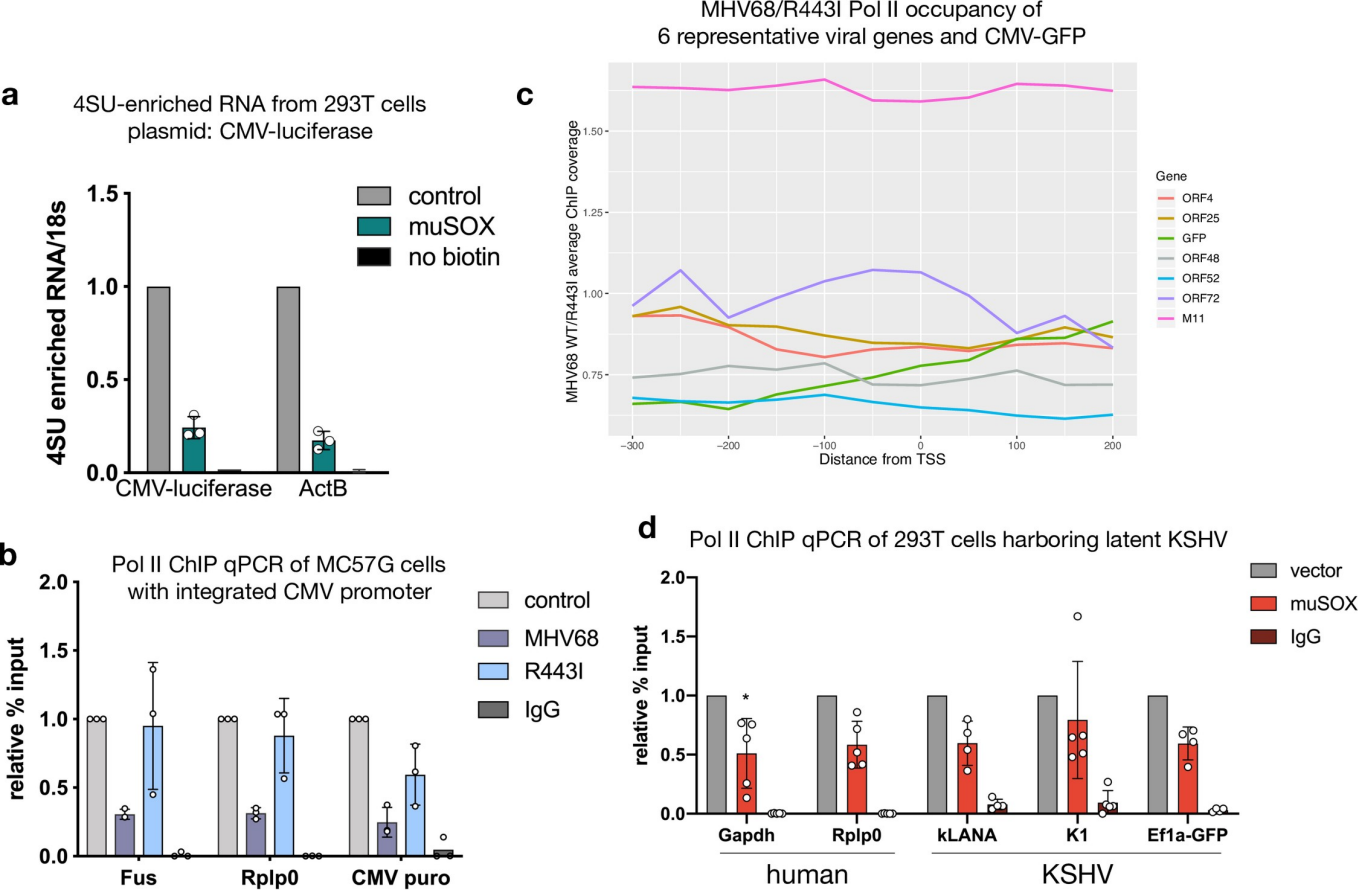
A non-MHV68 promoter escapes repression on the viral genome. (A) 293T cells were transfected with muSOX or a GFP control fused to the cell surface glycoprotein Thy1.1 with an intervening P2A ribosome skipping sequence. Pure populations of transfected cells were obtained by running cells over columns that enrich for Thy1.1 [23], whereupon 500 μM of 4SU was added for 10 min and labeled RNA was isolated by biotin-streptavidin pull down. Levels of newly transcribed RNA from the indicated genes were measured by RT-qPCR. All samples were normalized to 18S and levels of RNA from control GFP expressing cells were set to 1. (B) The CMV promoter driving puromycin resistance was lentivirally integrated into MC57G cells. The cells were then infected with WT MHV68 or R443I at an MOI of 5 and Pol II levels at the indicated promoters were assayed by Pol II ChIP-qPCR. (C) ChIP-seq traces comparing WT MHV68 to R443I coverage from -300 to +200 around the TSS in 25 bp bins, averaged from two biological replicates of infected MC57G cells. Six representative viral genes and CMV-GFP are shown. (D) 293T cells were infected with KSHV and then transfected with muSOX twice over a 24 h period to induce accelerated RNA turnover. Pol II levels at the indicated KSHV and human promoters were measured by ChIP-qPCR and plotted with standard deviation. (* p < 0.05, students t-test on raw % input values).

A prediction from the above results is that a non-MHV68 promoter would gain protection from transcriptional repression when present on the replicating viral genome. To test this, we measured the Pol II occupancy and transcriptional potential of the cytomegalovirus (CMV) promoter, which drives green fluorescent protein (GFP) expression on the MHV68 genome. Unlike alpha- and gammaherpesviruses, the betaherpesvirus CMV does not accelerate mRNA decay. Furthermore, in either a plasmid context or when integrated into 293T cells, the CMV promoter is strongly repressed during muSOX-induced mRNA decay, similar to host genes. This was seen upon measurement of nascent RNA production by 4SU pulse labeling in muSOX-expressing cells (Fig 7A, ActB shown for reference) [23]. We also observed repression of the CMV promoter driving puromycin resistance integrated into the host chromatin during infection by measuring Pol II occupancy using ChIP (Fig 7B). However, in the context of the replicating MHV68 genome, the CMV promoter driving GFP had a Pol II ChIP-seq signal during both MHV68 WT and R443I infection that was not markedly different from several other viral genes (ORFs 4, 25, 48, 52, 72, M11) (Fig 7C).

Finally, we evaluated whether the lytic stage of infection was necessary to confer escape from transcriptional repression, or whether some other feature of the non-replicating viral genome was important. To this end, we measured Pol II occupancy at promoters of several genes expressed from the KSHV genome during latent infection in 293T cells, when the viral DNA is maintained as an episome but not amplified in replication compartments. Latent infection does not promote mRNA turnover, and thus we transfected muSOX into these cells to stimulate mRNA turnover in the absence of lytic replication. Similar to the host genes *Gapdh* and *Rplp0*, we observed an RNA decay-induced repression of Pol II occupancy at the latent viral promoter LANA while the viral K1 promoter was repressed in 4 out of 5 experiments (Fig 7D). We also observed repression of the human *Ef1α* promoter, which is present on the KSHV viral episome as a driver of the GFP reporter gene (Fig 7D). In sum, these observations indicate that the viral genome per se does not confer protection from transcriptional repression; instead, features linked to lytic genome amplification in replication compartments facilitate robust Pol II recruitment under conditions of accelerated mRNA turnover.

## Discussion

Here, we demonstrate that virus-induced mRNA decay broadly decreases Pol II occupancy of mammalian promoters, extending prior observations made with individual cellular genes to the genomic scale. Using an unbiased TMT-based proteomics approach, we reveal that infection with MHV68 causes RNA decay-dependent changes in the abundance of numerous cellular proteins with roles in Pol II transcription, including depletion of 7 of the 12 Pol II subunits, all of which are specific to Pol II and not shared by Pol I or III. The TMT findings underscore how a multitude of alterations to protein localization and abundance likely shape the gene expression environment during infection, and further highlight the ripple effects of accelerating mRNA decay. Notably, promoters on the replicating viral genome recruit Pol II with similar efficiency during normal or accelerated mRNA decay, indicating that they escape transcriptional inhibition and overcome the decrease in Pol II and transcription factor subunits. This protection is lost upon relocation of the viral promoters into the host genome or when the viral genome is in a latent state, emphasizing the importance of DNA context and replication state, rather than promoter sequence, in determining the transcriptional response to infection.

We expect that the reduction in Pol II and general transcription factor protein levels during WT MHV68 infection at least partially underlies the ensuing Pol II occupancy defect on the host genome. Thus, at least two events contribute to mRNA decay-linked transcriptional repression: relocalization of RNA binding proteins such as PABPC to the nucleus upon degradation of muSOX-cleaved RNAs [23,25], and depletion of key transcriptional components during infection. The fact that knockdown of Xrn1 or Dis3L2 does not rescue Pol II occupancy during WT MHV68 infection indicates that either these mechanisms are redundant, or that the Pol II depletion phenotype is dominant. The mechanism underlying reduced Pol II occupancy upon protein relocalization remains unknown, but one hypothesis is that aberrant accumulation of RNA binding proteins in the nucleus is sensed as a cellular stress signal that causes the cell to reduce its gene expression [1,23]. While both phenotypes are observed during MHV68 infection, it is interesting that GTF and Pol II subunit depletion does not occur during muSOX-induced mRNA decay in the absence of infection [23]. Instead, other downstream effects of host shutoff, such as infection-specific relocalization of RBPs, or host shutoff dependent changes to viral protein levels, could result in these changes to a key category of proteins.

Pol II activity is also impeded during infection with some RNA viruses. For example, Influenza A virus reduces Pol II subunit levels through direct binding of Rpb1 by the viral polymerase, leading to decreased Pol II occupancy at host genes [38–41]. However, unlike herpesviruses, RNA viruses do not depend on Pol II for viral transcription. Efficient recruitment of Pol II to the MHV68 genome is notable given the decreased abundance of multiple Pol II subunits, which suggests that the viral genome out competes the host chromatin to attract the remaining pool of Pol II.

Across the host genome, Pol II appears to be most strongly affected at the stage of recruitment. This is supported by the TMT-MS data, which shows a decrease in the GTFs TFIIB and the large subunit of TFIIA that are key to formation of the preinitiation complex. The reduction in promoter-proximal Pol II is propagated into the gene body, although the differences there were less pronounced, suggesting that once transcription of a gene initiates there are not subsequent blocks to elongation. This agrees with prior studies of individual host loci in MHV68 infected or muSOX expressing cells [23,25]. The population of promoter-proximal Pol II detected by ChIP comprises a combination of actively engaged Pol II as well as Pol II transiently binding to the DNA. Thus, a large reduction in promoter-proximal Pol II may not be expected to be fully borne out in the gene body [42]. That said, we did note a robust decrease of Pol II in the *Rplp0* gene body of MHV68 infected cells when we used an antibody recognizing the N-terminus of Rpb1. It is therefore possible that the modest reduction in Pol II across gene bodies observed in the ChIP-seq using the 8WG16 antibody from MHV68 infected cells may underestimate the magnitude of the gene body decreases. We did not observe a defect in transcription termination, or evidence of polymerases on the DNA after TTSs, suggesting that MHV68 does not induce the termination defect and run-on transcription of host genes that occurs during infection with HSV-1 [43].

Reduced Pol II occupancy of the host genome during MHV68 infection parallels infection data from the alphaherpesvirus HSV-1. HSV-1 infection results in a genome-wide reduction in Pol II occupancy of host chromatin and reduced nascent RNA production of the majority of host transcripts [43–45]. Furthermore, early data showed that while the endogenous mouse β-globin locus was transcriptionally repressed during HSV-1 infection, its expression was rescued upon integration of the β-globin gene into the HSV-1 genome [46,47]. Experiments with gene-specific null viruses have implicated immediate early (IE) proteins in the HSV-1 transcriptional repression phenotype, including ICP27, which can accelerate mRNA turnover [45,48]. Thus, in addition to targeting host and viral mRNAs for cytoplasmic decay, both alpha and gammaherpesviruses restrict Pol II occupancy of the host genome, although additional work is needed to establish whether the underlying mechanism is similar.

During the lytic stage of herpesvirus infection, viral DNA is localized to replication compartments. These non-membrane bound structures are sites of viral genome replication, transcription, and packaging [49]. They are enriched for many host and viral proteins involved in these processes, including chromatin modifying factors and Pol II [34–36]. Replication compartments grow and coalesce during infection, excluding host chromatin, which becomes pushed to the nuclear periphery [31–33]. Previous analyses of cellular protein distribution by mass spectrometry revealed that muSOX and Xrn1-coordinated mRNA decay causes differential trafficking of many cellular RNA binding proteins between the cytoplasm and nucleus. At least one of these proteins, PABPC, was shown to be associated with transcriptional repression [23]. Here, in addition to observing increased PABPC in the nucleus of infected cells, we also detected PABPC in viral replication compartments. This indicates that while PABPC relocalizes to infected nuclei in a RNA decay-dependent manner, selective exclusion of PABPC from these compartments is not the mechanism by which the viral genome escapes repression. Instead, we hypothesize that either the state of viral DNA or other protective features of the replication compartment confers escape from transcriptional repression.

Protection from mRNA decay-induced transcriptional repression was not conferred to a non-MHV68 or viral promoters present on the latent viral episome, indicating that lytic phase viral DNA replication or replication compartment formation is key to this phenotype. The latent genomes of gammaherpesviruses including KSHV and Epstein Bar Virus (EBV) are heavily methylated and histone-rich, with the exception of promoters of genes expressed during latency, the latent origin of replication, and CTCF binding sites [50–52]. Notably, during latency key lytic promoters like RTA contain both the activating H3K4me3 and the repressive H3K27me3 histone marks, representing a ‘poised’ state [53]. Rapid changes shortly after lytic cycle induction result in hypomethylation and removal of repressive marks at early gene promoters [50,53]. At later stages of infection, KSHV, EBV and HSV-1 genomes have been shown to be largely nucleosome free, consistent with histones not being packaged in virions [33,35,54,55]. For gammaherpesviruses, this is the stage at which mRNA degradation is most pronounced. We therefore hypothesize that the ‘open’ state of viral DNA during lytic replication plays an important role in facilitating Pol II recruitment under conditions of accelerated cytoplasmic mRNA decay and may in fact be sufficient to explain the robust recruitment of Pol II to replication compartments. It is possible that the open state of the DNA, particularly late in infection, also contributes to the relatively uniform occupancy of Pol II across MHV68 genes and absence of clear promoter-proximal peaks. It will be interesting to investigate if the general chromatin state of the host genome also changes in infection or if is largely the lack of chromatinization of the viral genome that is driving these changes.

In conclusion, decay-coupled transcriptional repression is a novel facet of viral manipulation of the cellular gene expression landscape. We hypothesize that the ability to escape from transcriptional repression is linked to the open conformation of viral DNA in replication compartments, and that this helps these viruses counteract decay-induced viral transcript loss and overcome depletion of Pol II and general transcription factor protein subunits. Future work is geared towards exploring whether widespread mRNA decay alters cellular chromatin states and DNA accessibility and, if so, how this influences transcription factor recruitment.

## Materials and methods

### Plasmids and primers

pCDEF3 muSOX (Adgene 131695) and vhs (Addgene 131696) and SOX have been previously published [25]. pCDEF3-GFP was used as a control.

Primers used for qPCR and for cloning are listed in S2 Table. M7, M4 and CMV-puro were made by cloning the respective promoter sequence upstream of the puromycin cassette in a pLKO-sgRNA vector (Addgene 131697). The promoter of M4 was defined as the 1131 bp region upstream of the M4 coding region not overlapping with other viral ORFs and the promoter sequence of M7 was previously published [56]. pGL3-kLANA-puromycin (Addgene 131699) was made by introducing the puromycin gene into the I-CeuI and XbaI sites of pGL3-kLANA via InFusion (Clonetech) cloning. pGL3-kLANA was made by amplifying the KSHV LANA promoter sequence [57] and using InFusion cloning to introduce it into the XhoI and HindIII sites of pGL3.

### Cells and transfections

MC57G mouse fibroblast cells (ATCC), 293T cells (ATCC) and NIH 3T3 cells (ATCC) were maintained in DMEM (Invitrogen) supplemented with 10% fetal bovine serum (FBS). iSLK BAC16 cells [58] were maintained in DMEM supplemented with 10% FBS and 1 mg/mL hygromycin B. To reactivate iSLK cells, they were treated with 1 μg/mL doxycycline and 1 mM sodium butyrate for 48 hours.

DNA transfections were carried out in 293T cells at 70–90% confluency in 10 cm plates with 10 μg host-shutoff factor plasmid or vector control using PolyJet (SignaGen) 24 hours before harvesting and again at 18 hours before harvesting.

Lentiviral transduction was carried out by spinfecting 1x10^6^ 293T or MC57G cells with lentivirus made from 2^nd^ generation plasmids at a MOI <1 for 2 hours with 4 μg/mL polybrene (Fisher). Twenty-four hours later puromycin (1 μg/ml for 293T or 3 μg/ml for MC57G) was added to select for transduced cells. 293T cells stably expressing the kLANA-puromycin cassette were made by transfecting the cells with 1 μg pGL3-kLANA-puromycin using PolyJet, then selecting with puromycin for random integration events.

For the CMV-luciferase promoter assay, populations of 293T cells expressing muSOX or control GFP and the cell surface marker Thy1.1 were selected 24 hours post-transfection using the Miltenyi Biotec MACS cell separation system according to the manufacturer’s instructions as previously described [23]. Briefly, muSOX and GFP were each expressed from a vector containing an upstream Thy1.1 sequence separated from muSOX or GFP by a P2A ribosome skipping site, which results in separation of Thy1.1 from the downstream gene. Transfected cells were then passed over a column to enrich for Thy1.1-expressing cells, which were subsequently eluted and subjected to 4SU pulse labeling prior to harvesting.

293T cells were infected with KSHV by reactivating iSLK-Bac16 cells for 48 h and then spinfecting cell-free virus onto a monolayer of 293T cells, as described for lentiviral production but in the absence of polybrene.

3 million MC57G cells were nucleofected with a 100uL Neon tip (1400 Voltage/20 Width/2 Pulse) to knockdown Xrn1 or Dis3L2 using a pool of 4 siRNAs or a non-targeting control using 10 μL of 20 μM siRNA.

### 4SU labeling

Adapted from [59]. Cells were incubated in suspension with 500 μM 4SU (Sigma T4509) in DMEM supplemented with 10% FBS for 10 min then washed with PBS. RNA was extracted with TRIzol followed by isopropanol purification. RNA (300 μg) was incubated in biotinylation buffer (50 mM HEPES [pH 7.5], 5 mM EDTA) and 5 μg MTSEA-biotin (Biotium #90066), rotating at room temperature in the dark for 30 min. A no biotin control was made from an equal amount of total RNA and incubated as above but without the addition of MTSEA-biotin. RNA was then phenol:chloroform extracted and precipitated with isopropanol. The pellet was resuspended in DEPC-treated water and mixed with 50 μL Dynabeads MyOne streptavidin C1 (Invitrogen) that had been pre-washed twice with wash buffer (100 mM Tris [pH 7.5], 10 mM EDTA, 1 M NaCl, 0.1% Tween 20). Samples were rotated in the dark for 1 hour at room temperature, then washed twice with 65°C wash buffer and twice with room temperature wash buffer. Samples were eluted twice with 100 μL 5% Beta-mercaptoethanol (BME) in DEPC H_2_0 for 5 min then RNA was precipitated with isopropanol and quantified by RT-qPCR.

### Viral mutagenesis, propagation and infections

The WT and R443I MHV68 bacterial artificial chromosome (BAC) were previously described [7,60]. MHV68 was produced by first making p0 virus by transfecting NIH 3T3 cells in 6-well plates with 2.5 μg BAC DNA using TransIT-X2 (Mirus Bio) for 24h. Five to seven days later, the cells were split into a 10 cm dish, then harvested and frozen. After 5–7 days the majority of cells showed cytopathic effect (CPE). 30 μL of p0 was then added per confluent 10 cm dish of NIH 3T12 cells, split 2 days later into four 10 cm dishes and harvested 4–6 days later when all cells were infected. Cells were collected by centrifugation (5 min, 1500 rpm) and the pellet was dounced 10 times. The cell pellet and supernatant were then ultracentrifuged for 2 h at 30,000 rpm. The resulting pellet was resuspended and titered by plaque assay. Cells were infected with MHV68 at an MOI of 5 for 24 hours.

### Western blotting

Cells were lysed in RIPA buffer (50 mM Tris-HCl pH7.6, 150 mM NaCl, 3 mM MgCl_2_, 10% glycerol, 0.5% NP-40, cOmplete EDTA-free Protease Inhibitors [Roche]) and then clarified by centrifugation at 21,000 x g for 10 min at 4°C. Whole cell lysate was quantified by Bradford assay and resolved by SDS-PAGE. Antibodies for western blot are: Xrn1 (Bethyl A300-443A, 1:1000), Dis3L2 (Novus, NBP1 84740, 1:500), Rpb1 8WG16 (BioLegend, 1:500), Rpb2 (Santa Cruz sc-166803, 1:1000), TFIIB (Santa Cruz sc-271736, 1:1000), TBP (Abcam, ab51841, 1:1000) and Gapdh (Abcam, ab8245, 1:2000).

### RT-qPCR

RNA was reverse transcribed using AMV RT (Promega) with random 9-mer primers. Total RNA was DNase treated with TURBO DNase (ThermoFisher) or DNase treated on column with Zymo Direct-zol RNA MiniPrep Plus DNase. cDNA was quantified using iTaq Universal SYBR Mastermix (BioRad) and transcript-specific primers. All qPCR results are normalized to 18S levels and WT or vector control set to 1.

### Immunofluoresence

NIH 3T3 cells were plated on coverslips (7.5 x 10^4^ cells/well of a 12-well), infected the following day for 25–27 h, and then fixed in 4% formaldehyde for 10 min. iSLK-Bac16 cells were plated on coverslips (5 x 10^4^ cells/well of a 12-well), reactivated 24h later with doxycycline and sodium butyrate for 48 hours and then fixed as above. Cells were permeabilized with ice-cold methanol at -20°C for at least 20 min and incubated with anti-Pol II antibody (Biolegend, 8WG16 at 1:200), anti-PABPC antibody (Abcam ab21060 at 1:200 for mouse cells and Santa Cruz sc32318 at 1:25 for human cells) or anti-ORF59 antibody (Advanced Biotechnologies 13-211-100 at 1:200) in 5% BSA overnight at 4°C. Secondary antibodies were added (1:1000) for 1 h at 37°C. Coverslips were mounted in DAPI- containing Vectashield (VectorLabs). We identified RCs by Pol II recruitment or ORF59 staining corresponding with DAPI-poor regions [34,36,61]. Images were collected on a Zeiss LSM 710 AxioObserver with a 40x oil objective. Cells with RCs were first identified, then PABPC co-localization was determined by counting cells with at least 2x nuclear pixel intensity relative to non-RC containing cells in the same image (ImageJ).

### Chromatin immunoprecipitation (ChIP)

ChIP was performed from 15 cm plates of cells. 3 x 10^6^ MC57G or 293T cells were plated 24 h before infection and then harvested 24 h after infection or transfection(s). Upon harvesting, cells were washed with PBS, crosslinked in 1% formaldehyde at room temperature for 10 min. Cells were quenched in 0.125 M glycine for 5 min and washed twice with cold PBS. Crosslinked cell pellets were mixed with 1 mL fractionation buffer (5mM PIPES pH 8.0, 85 mM KCl, 0.5% NP-40 with cOmplete EDTA-free Protease Inhibitors [Roche]) and incubated on ice for 10 min, during which time the lysate was dounce homogenized to release nuclei and spun at 4,000 rpm for 5 min at 4°C to pellet nuclei. Nuclei were then resuspended in 300 μl of nuclei lysis buffer (50mM Tris-HCl pH 8.0, 0.3% SDS, 10mM EDTA, with cOmplete EDTA-free Protease Inhibitors [Roche]) and rotated for 10 min at 4°C followed by sonication using a QSonica Ultrasonicator. Sonicated chromatin was then spun at 13,000 rpm for 10 min at 4°C and the pellet discarded. 40 μg of chromatin measured with the Qubit broad range DNA reagent (ThermoFisher) was brought to 500 μL with ChIP dilution buffer (16.7 mM Tris-HCl pH 8.0, 1.1% Triton X-100, 1.2 mM EDTA, 167 mM NaCl and cOmplete EDTA-free Protease Inhibitors [Roche]) and incubated with 10 μg mouse monoclonal anti-RNAPII 8WG16 (BioLegend, used for all Pol II ChIP experiments unless indicated otherwise) or mouse monoclonal anti-RNAPII N-terminal domain (Cell Signaling14958S) or mouse IgG (Fisher Scientific) overnight, whereupon samples were rotated with 20 μl protein G dynabeads (Thermofisher) for 2 hours at 4°C. Beads were washed with low salt immune complex (20 mM Tris pH 8.0, 1% Triton-x-100, 2 mM EDTA, 150 mM NaCl, 0.1% SDS), high salt immune complex (20 mM Tris pH 8.0, 1% Triton-x-100, 2 mM EDTA, 500 mM NaCl, 0.1% SDS), lithium chloride immune complex (10 mM Tris pH 8.0, 0.25 M LiCl, 1% NP-40, 1% Deoxycholic acid, 1 mM EDTA), and Tris-EDTA for 5 min each at 4°C with rotation. DNA was eluted from the beads using 100 μl of elution buffer (150 mM NaCl, 50 μg/ml Proteinase K) and incubated at 55°C for 2 hours, then 65°C for 12 hours. DNA was purified using a Zymo Oligo Clean & Concentrator kit, and quantified by qPCR using primers to the promoter regions of indicated genes for 50 cycles. qPCR was simultaneously performed on input chromatin and normalized to input. Raw percent input values are normalized by setting mock infected or a transfection control to 1 and are plotted as relative % input. IgG pull downs were performed with the MHV68 infected sample under scramble conditions, or the muSOX or SOX transfected sample for transfection experiments.

### ChIP Sequencing and data analysis

Libraries were prepared for sequencing (Kapa Hyper Prep) using equal amounts of chromatin within each experiment, amplified for 10–12 cycles based on Kapa’s recommendations and sequenced on a HiSeq4000 with 100bp single end reads. Sequencing quality was assessed with FastQC and trimmed with Sickle. A custom index was made of the mm10 and MHV68 genomes with Bowtie2 build. Reads were mapped to that index using Bowtie2 (2.3.0).

To visualize reads in the Integrative Genomics Viewer (IGV), tdf files were made from bam files and indexes made from those bam files. To make tdf files for the viral genome, a chrom.size file was created for MHV68 and then the count command was run. Analysis of mapped reads was performed with HOMER (Hypergeometric Optimization of Motif EnRichment) [62]. Tag directories were created from the bam files generated from Bowtie with the makeTagDirectory command. This was done separately for the mouse and MHV68 genomes. For mouse genes, the analyzeRepeats command was used to look at tag density in the body of genes and the–c pausing option was used to compare pausing ratios. annotatePeaks was run in both the–hist and–ghist mode to assess composite Pol II occupancy across the mouse genome as well as individual gene occupancy.

Viral analyses were done by first creating a custom genome annotation in HOMER using the loadGenome command. The TSS file was created from data generously shared by Scott Tibbetts (University of Florida) including portions of the viral BAC provided by Laurie Krug (Stony Brook University). annotatePeaks was run in the–hist mode to assess composite Pol II occupancy across the viral genome as well as individual gene occupancy. Sequencing data are available on GEO repository (accession number GSE132574).

### Fractionation

NIH3T3 cells were fractionated into nuclear and cytoplasmic fractions using membrane filters as previously described [63]. Briefly, cells were washed twice with ice-cold PBS and the cell pellet was resuspended in 0.25 M sucrose, 25 mM KCl, 5 mM MgCl2, 20 mM HEPES-KOH, pH7.4 and incubated on ice for 10 min. The cells were lysed by passing them through a 14 μm polycarbonate membrane (Sterlitech Corporation). The nuclei were then isolated by centrifugation at 1,400 x *g* for 10 min and the supernatant was retained as the cytoplasmic fraction.

### Cell lysis, protein digestion and TMT labeling

Lysis, digestion, and TMT labeling were performed as previously described [23] with a few modifications. Briefly, nuclei were lysed in 100 mM Tris-HCl, pH 8.0, 4% SDS, 1 mM EDTA preheated to 70°C and cytoplasmic fractions were adjusted to 1% SDS. Complete lysis was achieved by successive rounds of heating and sonicating. After protein concentration assessment by BCA assay (Pierce), 100μg of protein from each sample was reduced and alkylated with 25 mM tris(2-carboxyethyl)phosphine (Pierce) and 50 mM chloroacetamide respectively and then the protein was precipitated via methanol-chloroform cleanup [64]. Samples were digested overnight at 37°C in 50 mM HEPES pH 8.3 at a 1:50 trypsin:protein ratio. Peptides from all three biological replicates were labeled with a 6-plex TMT kit following the scheme in Fig 3A and pooled in equal peptide amounts, resulting in three individual 6-plex experiments. Pooled peptides were fractionated by 2D StageTip as described [23] with the following changes: from the SCX fractionation, fractions 1 and 4 were pooled together and fractions 2 and 3 were pooled together prior to fractionation by SDB-RPS StageTips.

### LC-MS/MS and informatic analysis

Peptides were analyzed via LC-MS/MS using a Dionex Ultimate 3000 UPLC coupled online to an EASYSpray ion source and Q Exactive HF. A linear gradient of 5% ACN to 42% ACN in 0.1% FA over 150 min on an EASYSpray C18 column (75 μm x 50 cm) heated to 50°C was used to separate the peptides. Following ionization at 1.7kV, an MS1 scan was performed from 400 to 1800 m/z at 120,000 resolution with an automatic gain control (AGC) setting of 3e6 and a maximum injection time (MIT) of 30 ms recorded in profile. The top 15 precursors were then selected for fragmentation and MS2 scans were acquired at a resolution of 30,000 with an AGC setting of 1e5, a MIT of 42 ms, an isolation window of 0.8 m/z, a fixed first mass of 100 m/z, normalized collision energy of 33, intensity threshold of 2e5, peptide match set to preferred, and a dynamic exclusion of 45 s recorded in profile.

MS/MS data were analyzed by Proteome Discoverer (Thermo Fisher Scientific, v2.2.0.388). The nuclear channels (126 – 128) and cytoplasmic channels (129–131) were analyzed in separate Proteome Discoverer studies as described before [23]. A fully tryptic search against a mouse Uniprot database appended with MHV68 and common contaminant sequences (downloaded 10/2016–17,149 sequences) requiring 5 ppm mass accuracy on the precursor ions and 0.02 Da accuracy on the fragment ions was performed. Static carbamidomethyl modifications to cysteine, static TMT additions to peptide N-termini and lysine residues, dynamic oxidation of methionine, dynamic deamidation of asparagine, dynamic methionine loss and acetylation of protein n-termini, and dynamic phosphorylation of serine, threonine, and tyrosine were allowed as modifications in the search. Matched spectra were scored by Percolator and reporter ion signal-to-noise values were extracted. Following parsimonious protein assembly at a 1% FDR for proteins and peptides, reporter ion quantitation was performed for unique and razor peptides with an average S/N of at least 10 and a precursor co-isolation threshold of less than 30% which did not contain a variable modification and normalized to the total detected signal in each TMT channel. Protein abundances were calculated as the sum of all reporter ion values in a particular channel for each protein. Imputation of missing values was performed by low abundance resampling. The data were scaled based on the Mock infection samples (channels 126 and 129). Statistically differential proteins were assessed via the background based t-test analysis implemented in Proteome Discoverer. The resulting data was exported to Excel for further analysis. The mass spectrometry proteomics data reported in this paper have been deposited at the ProteomeXchange Consortium via the PRIDE partner repository [65]. The PRIDE accession number is PXD015786.

## Supporting information

S1 Fig ChIP-seq replicate with WT and R443I MHV68. A) Pol II ChIP-seq signal profiles of host genes are shown in mock-infected, MHV68-infected and R443I-infected cells. Each row of the heat map displays Pol II occupancy of one gene from -1000 to +1000 in 25 bp bins. Genes are ranked by the Pol II-transcription start site (TSS) proximal signal in mock infected cells. (B) Sequence tags were plotted as a histogram in 25 bp bins for -2000 to +4000 around the TSS. Mock, MHV68 (red) and MHV68 (blue) and R443I (green) traces are shown along with their input controls. (C) ChIP-qPCR validation of Pol II occupancy at the *Rplp0* and *Fus* promoters using an antibody specific for the N-terminus of Rpb1 plotted with standard deviation. Pol II ChIP was performed on mock, MHV68 WT or MHV68 R443I infected MC57G cells and Pol II levels were assayed near the TSS of two repressed host genes during MHV68 infection from the ChIP-seq data. IgG is from the MHV68 infection condition. (* p < 0.05, ** p < 0.001, students paired t-test on raw % input values) (D) Pol II transcription termination is not dependent on RNA decay. Sequence tags were plotted as a histogram in 25 bp bins for transcription termination sequence (TTS) proximal Pol II for -1000 to +1000 around the TTS with the same color scheme as (B).
(TIF)

S2 Fig TMT-MS fractionation validation and Panther DB terms broken down by cellular compartment and condition. A) Reporter ion abundance from the TMT-MS data showing that the nuclear and cytoplasmic distribution of the nuclear protein H4 and the cytoplasmic protein GAPDH are primarily detected in their correct compartments, demonstrating successful fractionation. Graphs display the mean with standard deviation of 9 biological replicates including mock, MHV68 and R443I infection conditions. (B-D) Gene ontology terms for proteins increased and decreased in each compartment in a host shutoff dependent manner. Lists were generated by taking all proteins with a log 2 fold change greater than 0.2 comparing WT MHV68 to R443I and looking at the molecular function enrichment in Panther DB [66]. Terms with fold enrichment greater than 6 were included for the cytoplasm and greater than 5 for the nucleus.
(TIF)

S3 Fig 4SU-enriched RNA from MHV68 and R443I infected MC57G cells. MC57G cells were infected with WT or R443I MHV68 for 24 h, whereupon 500 μM of 4sU was added for 10 min and labeled RNA was isolated by biotin-streptavidin pull down. Levels of newly transcribed RNA from the indicated viral genes were measured by RT-qPCR. All samples were normalized to 18S and R443I-infected levels set to 1.
(TIF)

S4 Fig Unreactivated iSLK cells show primarily cytoplasmic PABPC signal. An immunofluorescence assay was performed on unreactivated (latent) KSHV-positive iSLK cells using antibodies against PABPC and the viral lytic protein ORF59. DNA was stained with DAPI.
(TIF)

S1 Table Reporter ion abundances from TMT-MS of NIH3T3 mouse fibroblasts infected with WT MHV68 or R443I MHV68. NIH3T3 mouse fibroblasts were infected with WT MHV68 or R443I MHV68 then fractionated into nucleus and cytoplasm, labeled with tandem mass tags and analyzed by quantitative liquid chromatography/mass spectrometry. This table provides protein identification information, normalized and scaled reporter ion abundances for each compartment.
(XLSX)

S2 Table List of all DNA sequences used in this study. (XLSX)

## References

[1] Hartenian E, Glaunsinger BA. Feedback to the central dogma: cytoplasmic mRNA decay and transcription are interdependent processes. Critical Reviews in Biochemistry and Molecular Biology. 2019 10 16;0(0):1–14. 10.1080/10409238.2019.1679083 PMC6871655 31656086

[2] Sun M, Schwalb B, Schulz D, Pirkl N, Etzold S, Lariviere L, et al Comparative dynamic transcriptome analysis (cDTA) reveals mutual feedback between mRNA synthesis and degradation. Genome Research. 2012 7 2;22(7):1350–9. 10.1101/gr.130161.111 22466169 PMC3396375

[3] Haimovich G, Medina DA, Causse SZ, Garber M, Millán-Zambrano G, Barkai O, et al Gene Expression Is Circular: Factors for mRNA Degradation Also Foster mRNA Synthesis. Cell. 2013 5 23;153(5):1000–11. 10.1016/j.cell.2013.05.012 23706738

[4] Shalem O, Dahan O, Levo M, Martinez MR, Furman I, Segal E, et al Transient transcriptional responses to stress are generated by opposing effects of mRNA production and degradation. Mol Syst Biol. 2008;4(1):223–10. 18854817 10.1038/msb.2008.59 PMC2583085

[5] Shalem O, Groisman B, Choder M, Dahan O, Pilpel Y. Transcriptome Kinetics Is Governed by a Genome-Wide Coupling of mRNA Production and Degradation: A Role for RNA Pol II. Barsh GS, editor. 2011 9 8;7(9):e1002273–10. 10.1371/journal.pgen.1002273 21931566 PMC3169527

[6] Dori-Bachash M, Shalem O, Manor YS, Pilpel Y, Tirosh I. Widespread promoter-mediated coordination of transcription and mRNA degradation. Genome Biol. 2012;13(12). 10.1186/gb-2012-13-12-r114 PMC4056365 23237624

[7] Richner JM, Clyde K, Pezda AC, Cheng BYH, Wang T, Kumar GR, et al Global mRNA Degradation during Lytic Gammaherpesvirus Infection Contributes to Establishment of Viral Latency. Speck SH, editor. PLoS Pathog. 2011 7 21;7(7):e1002150–13. 10.1371/journal.ppat.1002150 21811408 PMC3141057

[8] Liu SW, Wyatt LS, Orandle MS, Minai M, Moss B. The D10 Decapping Enzyme of Vaccinia Virus Contributes to Decay of Cellular and Viral mRNAs and to Virulence in Mice. Journal of Virology. 6 ed. 2014 1 1;88(1):202–11. 10.1128/JVI.02426-13 24155373 PMC3911708

[9] Parrish S, Moss B. Characterization of a vaccinia virus mutant with a deletion of the D10R gene encoding a putative negative regulator of gene expression. Journal of Virology. 2006 1;80(2):553–61. 10.1128/JVI.80.2.553-561.2006 16378957 PMC1346865

[10] Read GS. Virus-encoded endonucleases: expected and novel functions. WIREs RNA. 1st ed. 2013 7 30;4(6):693–708. 10.1002/wrna.1188 23900973

[11] Dauber B, Poon D, Santos dos T, Duguay BA, Mehta N, Saffran HA, et al The Herpes Simplex Virus Virion Host Shutoff Protein Enhances Translation of Viral True Late mRNAs Independently of Suppressing Protein Kinase R and Stress Granule Formation. Sandri-Goldin RM, editor. Journal of Virology. 2016 6 10;90(13):6049–57. 10.1128/JVI.03180-15 27099317 PMC4907241

[12] Karr BM, Read GS. The virion host shutoff function of herpes simplex virus degrades the 5’ end of a target mRNA before the 3’ end. Virology. 1999 11 10;264(1):195–204. 10.1006/viro.1999.9986 10544145

[13] Glaunsinger B, Chavez L, Ganem D. The Exonuclease and Host Shutoff Functions of the SOX Protein of Kaposi's Sarcoma-Associated Herpesvirus Are Genetically Separable. Journal of Virology. 2005 5 26;79(12):7396–401. 10.1128/JVI.79.12.7396-7401.2005 15919895 PMC1143623

[14] Huang C, Lokugamage KG, Rozovics JM, Narayanan K, Semler BL, Makino S. SARS Coronavirus nsp1 Protein Induces Template-Dependent Endonucleolytic Cleavage of mRNAs: Viral mRNAs Are Resistant to nsp1-Induced RNA Cleavage. Baric RS, editor. PLoS Pathog. 2011 12 8;7(12):e1002433–18. 10.1371/journal.ppat.1002433 22174690 PMC3234236

[15] Kamitani W, Huang C, Narayanan K, Lokugamage KG, Makino S. A two-pronged strategy to suppress host protein synthesis by SARS coronavirus Nsp1 protein. Nature Structural & Molecular Biology. 2009 10 18;16(11):1134–40. 10.1038/nsmb.1680 PMC2784181 19838190

[16] Jagger BW, Wise HM, Kash JC, Walters K-A, Wills NM, Xiao Y-L, et al An Overlapping Protein-Coding Region in Influenza A Virus Segment 3 Modulates the Host Response. Science. 2012;337(6091):199–204. 10.1126/science.1222213 22745253 PMC3552242

[17] Covarrubias S, Gaglia MM, Kumar GR, Wong W, Jackson AO, Glaunsinger BA. Coordinated Destruction of Cellular Messages in Translation Complexes by the Gammaherpesvirus Host Shutoff Factor and the Mammalian Exonuclease Xrn1. Renne R, editor. PLoS Pathog. 2011 10 27;7(10):e1002339–15. 10.1371/journal.ppat.1002339 22046136 PMC3203186

[18] Gaglia MM, Covarrubias S, Wong W, Glaunsinger BA. A Common Strategy for Host RNA Degradation by Divergent Viruses. Journal of Virology. 2012 8 9;86(17):9527–30. 10.1128/JVI.01230-12 22740404 PMC3416159

[19] Eckmann CR, Rammelt C, Wahle E. Control of poly(A) tail length. WIREs RNA. 2010 11 17;2(3):348–61. 10.1002/wrna.56 21957022

[20] Gallouzi IE, Wilusz J. A DIStinctively novel exoribonuclease that really likes U. EMBO J. 2013 6 11;32(13):1799–801. 10.1038/emboj.2013.136 23756464 PMC3981174

[21] Schoenberg DR, Maquat LE. Re-capping the message. Trends in Biochemical Sciences. 2009 9;34(9):435–42. 10.1016/j.tibs.2009.05.003 19729311 PMC2743798

[22] Abernathy E, Glaunsinger B. Emerging roles for RNA degradation in viral replication and antiviral defense. Virology. 2015 5 1;479-480(C):600–8. 25721579 10.1016/j.virol.2015.02.007 PMC4424162

[23] Gilbertson S, Federspiel JD, Hartenian E, Cristea I, Glausninger B. Changes in mRNA abundance drive shuttling of RNA binding proteins, linking cytoplasmic RNA degradation to transcription. eLife. 2018. 10.7554/eLife.37663 PMC6203436 30281021

[24] Abernathy E, Clyde K, Yeasmin R, Krug LT, Burlingame A, Coscoy L, et al Gammaherpesviral Gene Expression and Virion Composition Are Broadly Controlled by Accelerated mRNA Degradation. van Dyk LF, editor. PLoS Pathog. 2014 1 16;10(1):e1003882–14. 10.1371/journal.ppat.1003882 24453974 PMC3894220

[25] Abernathy E, Gilbertson S, Alla R, Glaunsinger B. Viral Nucleases Induce an mRNA Degradation- Transcription Feedback Loop in Mammalian Cells. Cell Host Microbe. 2015 7 21;18(2):1–12. 26211836 10.1016/j.chom.2015.06.019 PMC4538998

[26] Martinez-Guzman D, Rickabaugh T, Wu T-T, Brown H, Cole S, Song MJ, et al Transcription program of murine gammaherpesvirus 68. Journal of Virology. 2003 10;77(19):10488–503. 10.1128/JVI.77.19.10488-10503.2003 12970434 PMC228380

[27] Nojima T, Gomes T, Grosso ARF, Kimura H, Dye MJ, Dhir S, et al Mammalian NET-Seq Reveals Genome-wide Nascent Transcription Coupled to RNA Processing. Cell. 2015 4 23;161(3):526–40. 10.1016/j.cell.2015.03.027 25910207 PMC4410947

[28] Glaunsinger B, Ganem D. Lytic KSHV infection inhibits host gene expression by accelerating global mRNA turnover. Molecular Cell. 2004;13(5):713–23. 10.1016/s1097-2765(04)00091-7 15023341

[29] Rodriguez W, Srivastav K, Muller M. C19ORF66 Broadly Escapes Virus-Induced Endonuclease Cleavage and Restricts Kaposi's Sarcoma-Associated Herpesvirus. Jung JU, editor. Journal of Virology. 2019 6 15;93(12):9527–12. 10.1128/JVI.00373-19 PMC6613750 30944177

[30] Clyde K, Glaunsinger BA. Deep Sequencing Reveals Direct Targets of Gammaherpesvirus-Induced mRNA Decay and Suggests That Multiple Mechanisms Govern Cellular Transcript Escape. Means RE, editor. PLoS ONE. 2011 5 9;6(5):e19655–12. 10.1371/journal.pone.0019655 21573023 PMC3090416

[31] Chang L, Godinez WJ, the IKPO, 2011 Herpesviral replication compartments move and coalesce at nuclear speckles to enhance export of viral late mRNA. Proceedings of the National Academy of Sciences. 10.1073/pnas.1103411108 PMC3102408 21555562

[32] Taylor TJ, McNamee EE, Day C, Knipe DM. Herpes simplex virus replication compartments can form by coalescence of smaller compartments. Virology. 2003 5;309(2):232–47. 10.1016/s0042-6822(03)00107-7 12758171

[33] Monier K, Armas JCG, Etteldorf S, Ghazal P, Sullivan KF. Annexation of the interchromosomal space during viral infection. Nat Cell Biol. 2000 9 1;2(9):661–5. 10.1038/35023615 10980708

[34] Li D, Fu W, Swaminathan S. Continuous DNA replication is required for late gene transcription and maintenance of replication compartments in gammaherpesviruses. Robertson ES, editor. PLoS Pathog. 2018 5 29;14(5):e1007070–25. 10.1371/journal.ppat.1007070 29813138 PMC5993329

[35] Dembowski JA, DeLuca NA. Selective Recruitment of Nuclear Factors to Productively Replicating Herpes Simplex Virus Genomes. Everett RD, editor. PLoS Pathog. 2015 5 27;11(5):e1004939–35. 10.1371/journal.ppat.1004939 26018390 PMC4446364

[36] Rice SA, Long MC, LAM V, Spencer CA. Rna-Polymerase-Ii Is Aberrantly Phosphorylated and Localized to Viral Replication Compartments Following Herpes-Simplex Virus-Infection. Journal of Virology. 1994 2;68(2):988–1001. 8289400 10.1128/jvi.68.2.988-1001.1994 PMC236537

[37] Kumar GR, Shum L, Glaunsinger BA. Importin alpha-mediated nuclear import of cytoplasmic poly(A) binding protein occurs as a direct consequence of cytoplasmic mRNA depletion. Molecular and Cellular Biology. 2011 8;31(15):3113–25. 10.1128/MCB.05402-11 21646427 PMC3147611

[38] Ahmed M, Lyles DS. Effect of vesicular stomatitis virus matrix protein on transcription directed by host RNA polymerases I, II, and III. Journal of Virology. 1998 10;72(10):8413–9. 9733895 10.1128/jvi.72.10.8413-8419.1998 PMC110232

[39] Verbruggen P, Ruf M, Blakqori G, Överby AK, Heidemann M, Eick D, et al Interferon Antagonist NSs of La Crosse Virus Triggers a DNA Damage Response-like Degradation of Transcribing RNA Polymerase II. Journal of Biological Chemistry. 2011 1 28;286(5):3681–92. 10.1074/jbc.M110.154799 21118815 PMC3030371

[40] Vreede FT, Chan AY, Sharps J, Fodor E. Mechanisms and functional implications of the degradation of host RNA polymerase II in influenza virus infected cells. Virology. 2010 1 5;396(1):125–34. 10.1016/j.virol.2009.10.003 19875144 PMC2791857

[41] Black BL, Lyles DS. Vesicular stomatitis virus matrix protein inhibits host cell-directed transcription of target genes in vivo. Journal of Virology. 1992 7;66(7):4058–64. 1318397 10.1128/jvi.66.7.4058-4064.1992 PMC241208

[42] Darzacq X, Shav-Tal Y, de Turris V, Brody Y, Shenoy SM, Phair RD, et al In vivo dynamics of RNA polymerase II transcription. Nat Struct Mol Biol. 2007 8 5;14(9):796–806. 10.1038/nsmb1280 17676063 PMC4942130

[43] Rutkowski AJ, Erhard F, L'Hernault A, Bonfert T, Schilhabel M, Crump C, et al Widespread disruption of host transcription termination in HSV-1 infection. Nature Communications. 2015;6:7126 10.1038/ncomms8126 25989971 PMC4441252

[44] Abrisch RG, Eidem TM, Yakovchuk P, Kugel JF, Goodrich JA. Infection by Herpes Simplex Virus 1 Causes Near-Complete Loss of RNA Polymerase II Occupancy on the Host Cell Genome. Sandri-Goldin RM, editor. Journal of Virology. American Society for Microbiology; 2016 3;90(5):2503–13. 10.1128/JVI.02665-15 PMC4810688 26676778

[45] Spencer CA, Dahmus ME, Rice SA. Repression of host RNA polymerase II transcription by herpes simplex virus type 1. Journal of Virology. 1997 3;71(3):2031–40. 9032335 10.1128/jvi.71.3.2031-2040.1997 PMC191289

[46] Smiley JR, Smibert C, Everett RD. Expression of a Cellular Gene Cloned in Herpes-Simplex Virus—Rabbit Beta-Globin Is Regulated as an Early Viral Gene in Infected Fibroblasts. Journal of Virology. 1987 8;61(8):2368–77. 3037101 10.1128/jvi.61.8.2368-2377.1987 PMC255648

[47] Smibert CA, Smiley JR. Differential Regulation of Endogenous and Transduced Beta-Globin Genes During Infection of Erythroid-Cells with a Herpes-Simplex Virus Type-1 Recombinant. Journal of Virology. 1990 8;64(8):3882–94. 1695257 10.1128/jvi.64.8.3882-3894.1990 PMC249684

[48] Smiley JR. Herpes simplex virus virion host shutoff protein: immune evasion mediated by a viral RNase? Journal of Virology. 2004 2;78(3):1063–8. 10.1128/JVI.78.3.1063-1068.2004 14722261 PMC321390

[49] Schmid M, Speiseder T, Dobner T, Gonzalez RA. DNA Virus Replication Compartments. Journal of Virology. 4 ed. 2014 1 14;88(3):1404–20. 10.1128/JVI.02046-13 24257611 PMC3911613

[50] Günther T, Grundhoff A. The Epigenetic Landscape of Latent Kaposi Sarcoma-Associated Herpesvirus Genomes. Kellam P, editor. PLoS Pathog. 2010 6 3;6(6):e1000935–19. 10.1371/journal.ppat.1000935 20532208 PMC2880564

[51] Szyf M, Eliasson L, Mann V, Klein G, Razin A. Cellular and Viral-DNA Hypomethylation Associated with Induction of Epstein-Barr Virus Lytic Cycle. Proc Natl Acad Sci USA. 1985;82(23):8090–4. 10.1073/pnas.82.23.8090 2999791 PMC391448

[52] Hilton IB, Simon JM, Lieb JD, Davis IJ, Damania B, Dittmer DP. The Open Chromatin Landscape of Kaposi's Sarcoma-Associated Herpesvirus. Journal of Virology. 2013 10 3;87(21):11831–42. 10.1128/JVI.01685-13 23986576 PMC3807352

[53] Toth Z, Maglinte DT, Lee SH, Lee H-R, Wong L-Y, Brulois KF, et al Epigenetic Analysis of KSHV Latent and Lytic Genomes. Kellam P, editor. PLoS Pathog. 2010 7 22;6(7):e1001013–7. 10.1371/journal.ppat.1001013 20661424 PMC2908616

[54] Hollingworth R, Horniblow RD, Forrest C, Stewart GS, Grand RJ. Localization of Double-Strand Break Repair Proteins to Viral Replication Compartments following Lytic Reactivation of Kaposi's Sarcoma-Associated Herpesvirus. Jung JU, editor. Journal of Virology. 2017 10 27;91(22):1186–21. 10.1128/JVI.00930-17 PMC5660498 28855246

[55] McSwiggen DT, Hansen AS, Teves SS, et al 2019 Evidence for DNA-mediated nuclear compartmentalization distinct from phase separation. eLife. 10.7554/eLife.47098 PMC6522219 31038454

[56] Wong-Ho E, Wu TT, Davis ZH, Zhang B, Huang J, Gong H, et al Unconventional Sequence Requirement for Viral Late Gene Core Promoters of Murine Gammaherpesvirus 68. Journal of Virology. 2014 2 24;88(6):3411–22. 10.1128/JVI.01374-13 24403583 PMC3957950

[57] Jeong J, Papin J, Dittmer D. Differential Regulation of the Overlapping Kaposi's Sarcoma-Associated Herpesvirus vGCR (orf74) and LANA (orf73) Promoters. Journal of Virology. 2001 2 15;75(4):1798–807. 10.1128/JVI.75.4.1798-1807.2001 11160678 PMC114089

[58] Brulois KF, Chang H, Lee ASY, Ensser A, Wong LY, Toth Z, et al Construction and Manipulation of a New Kaposi's Sarcoma-Associated Herpesvirus Bacterial Artificial Chromosome Clone. Journal of Virology. 2012 8 23;86(18):9708–20. 10.1128/JVI.01019-12 22740391 PMC3446615

[59] Duffy EE, Rutenberg-Schoenberg M, Stark CD, Kitchen RR, Gerstein MB, Simon MD. Tracking Distinct RNA Populations Using Efficient and Reversible Covalent Chemistry. Molecular Cell. 2015 9;59(5):858–66. 10.1016/j.molcel.2015.07.023 26340425 PMC4560836

[60] Adler H, Messerle M, Wagner M, Koszinowski UH. Cloning and mutagenesis of the murine gammaherpesvirus 68 genome as an infectious bacterial artificial chromosome. Journal of Virology. 2000 8;74(15):6964–74. 10.1128/jvi.74.15.6964-6974.2000 10888635 PMC112213

[61] Dai-Ju JQ, Li L, Johnson LA, Sandri-Goldin RM. ICP27 Interacts with the C-Terminal Domain of RNA Polymerase II and Facilitates Its Recruitment to Herpes Simplex Virus 1 Transcription Sites, Where It Undergoes Proteasomal Degradation during Infection. Journal of Virology. 2006 3 14;80(7):3567–81. 10.1128/JVI.80.7.3567-3581.2006 16537625 PMC1440381

[62] Heinz S, Benner C, Spann N, Bertolino E, Lin YC, Laslo P, et al Simple Combinations of Lineage-Determining Transcription Factors Prime cis-Regulatory Elements Required for Macrophage and B Cell Identities. Molecular Cell. 2010 5 28;38(4):576–89. 10.1016/j.molcel.2010.05.004 20513432 PMC2898526

[63] Beltran PMJ, Mathias RA, Cristea IM. A Portrait of the Human Organelle Proteome In Space and Time during Cytomegalovirus Infection. Cell Systems. 2016 10 26;3(4):361–6. 10.1016/j.cels.2016.08.012 27641956 PMC5083158

[64] Wessel D, Flügge UI. A method for the quantitative recovery of protein in dilute solution in the presence of detergents and lipids. Anal Biochem. 1984 4;138(1):141–3. 10.1016/0003-2697(84)90782-6 6731838

[65] Vizcaíno JA, Côté RG, Csordas A, Dianes JA, Fabregat A, Foster JM, et al The Proteomics Identifications (PRIDE) database and associated tools: status in 2013. Nucleic Acids Res. 2012 11 29;41(D1):D1063–9. 23203882 10.1093/nar/gks1262 PMC3531176

[66] Mi H, Muruganujan A, Ebert D, Huang X, Thomas PD. PANTHER version 14: more genomes, a new PANTHER GO-slim and improvements in enrichment analysis tools. Nucleic Acids Res. Oxford University Press; 2018 11 8;47(D1):D419–26. 10.1093/nar/gky1038 PMC6323939 30407594

