# Supplementary figures and images for: RNA decay during gammaherpesvirus infection reduces RNA polymerase II occupancy of host promoters but spares viral promoters
Source: PLoS Pathog. 2020 Feb 7;16(2):e1008269. doi: 10.1371/journal.ppat.1008269 (PMC7032723; doi:10.1371/journal.ppat.1008269)

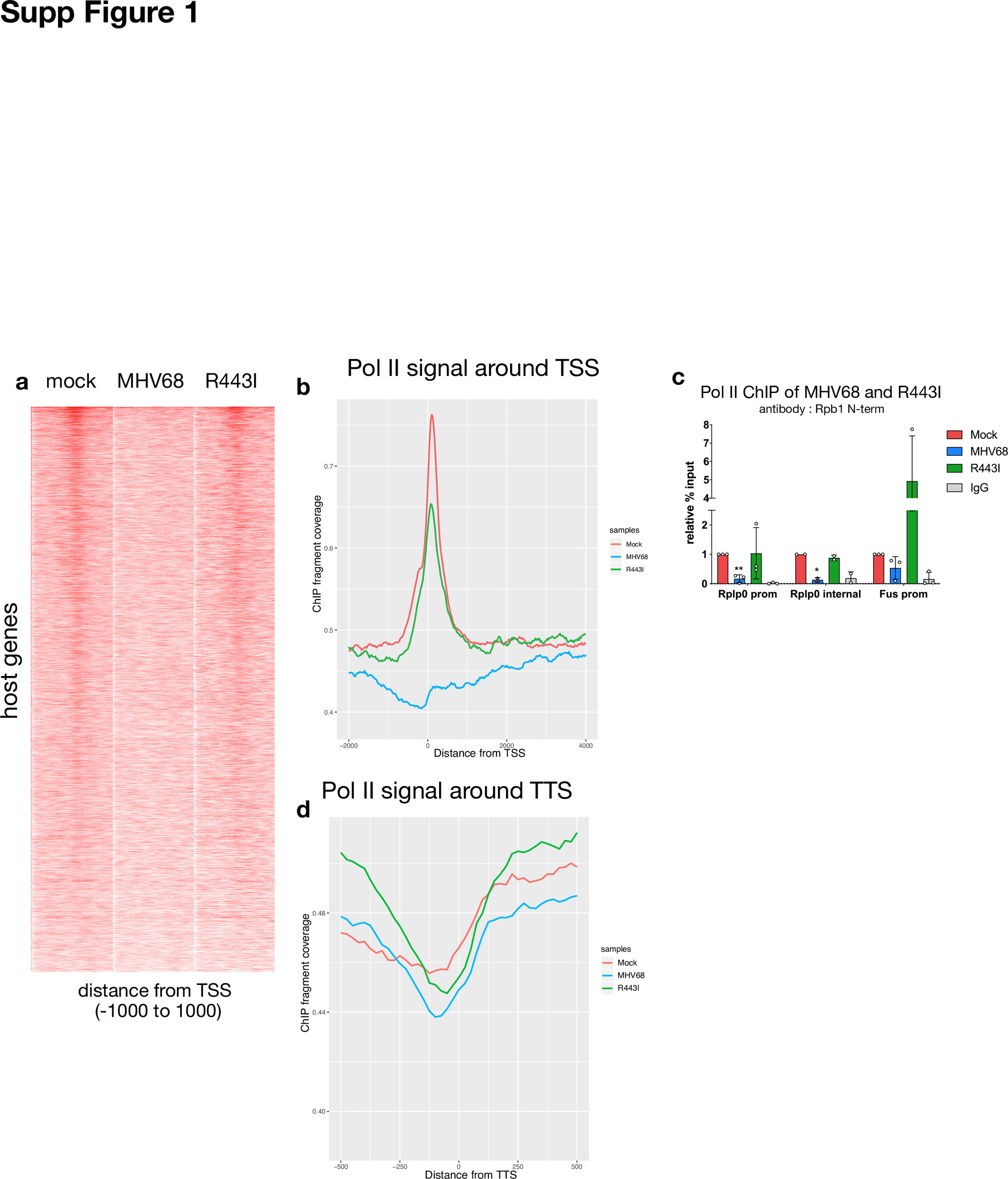

Supplement: S1 Fig — A) Pol II ChIP-seq signal profiles of host genes are shown in mock-infected, MHV68-infected and R443I-infected cells. Each row of the heat map displays Pol II occupancy of one gene from -1000 to +1000 in 25 bp bins. Genes are ranked by the Pol II-transcription start site (TSS) proximal signal in mock infected cells. (B) Sequence tags were plotted as a histogram in 25 bp bins for -2000 to +4000 around the TSS. Mock, MHV68 (red) and MHV68 (blue) and R443I (green) traces are shown along with their input controls. (C) ChIP-qPCR validation of Pol II occupancy at the Rplp0 and Fus promoters using an antibody specific for the N-terminus of Rpb1 plotted with standard deviation. Pol II ChIP was performed on mock, MHV68 WT or MHV68 R443I infected MC57G cells and Pol II levels were assayed near the TSS of two repressed host genes during MHV68 infection from the ChIP-seq data. IgG is from the MHV68 infection condition. (* p < 0.05, ** p < 0.001, students paired t-test on raw % input values) (D) Pol II transcription termination is not dependent on RNA decay. Sequence tags were plotted as a histogram in 25 bp bins for transcription termination sequence (TTS) proximal Pol II for -1000 to +1000 around the TTS with the same color scheme as (B). (TIF) [file ppat.1008269.s001.tif]

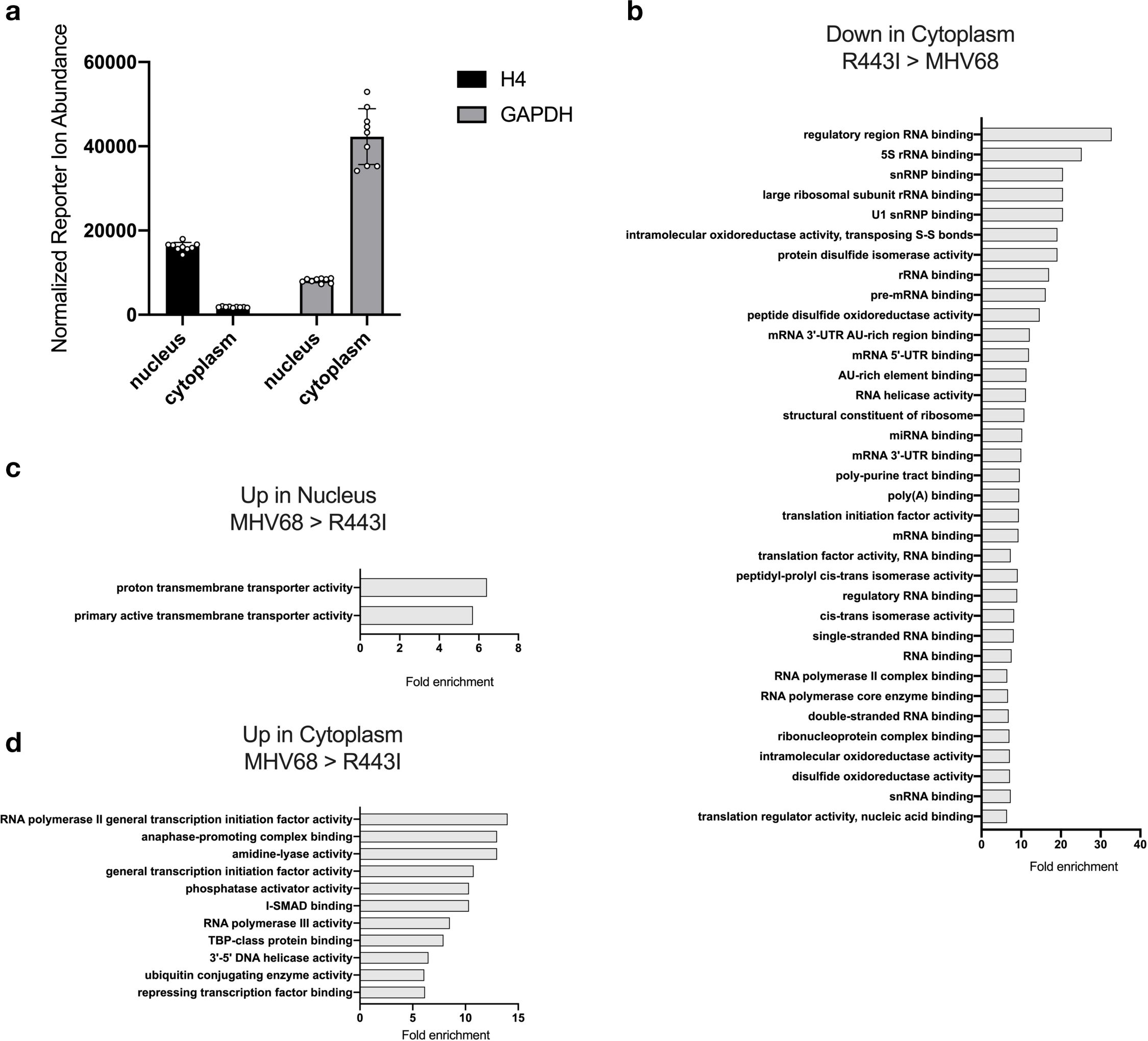

Supplement: S2 Fig — A) Reporter ion abundance from the TMT-MS data showing that the nuclear and cytoplasmic distribution of the nuclear protein H4 and the cytoplasmic protein GAPDH are primarily detected in their correct compartments, demonstrating successful fractionation. Graphs display the mean with standard deviation of 9 biological replicates including mock, MHV68 and R443I infection conditions. (B-D) Gene ontology terms for proteins increased and decreased in each compartment in a host shutoff dependent manner. Lists were generated by taking all proteins with a log 2 fold change greater than 0.2 comparing WT MHV68 to R443I and looking at the molecular function enrichment in Panther DB [66]. Terms with fold enrichment greater than 6 were included for the cytoplasm and greater than 5 for the nucleus. (TIF) [file ppat.1008269.s002.tif]

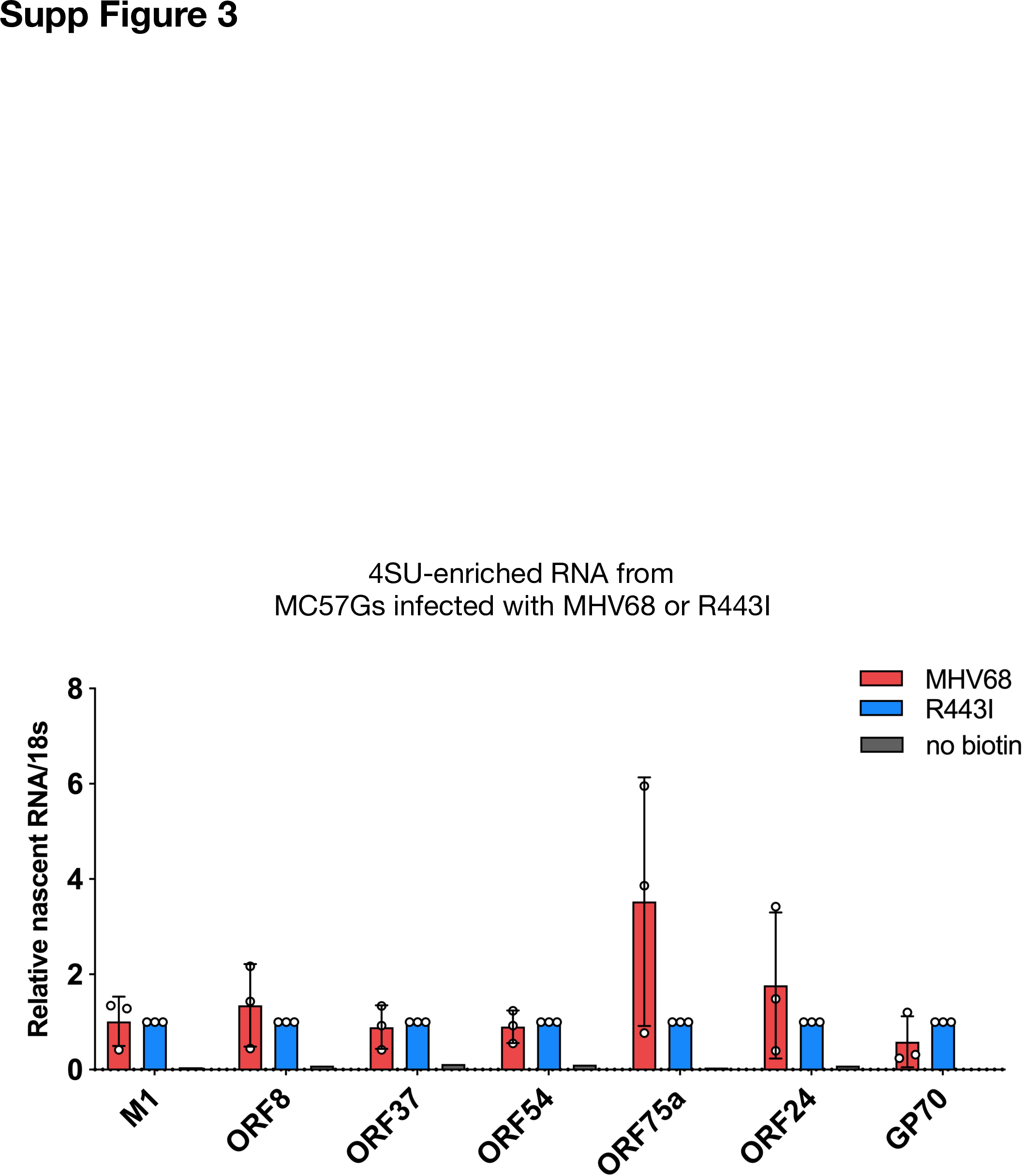

Supplement: S3 Fig — MC57G cells were infected with WT or R443I MHV68 for 24 h, whereupon 500 μM of 4sU was added for 10 min and labeled RNA was isolated by biotin-streptavidin pull down. Levels of newly transcribed RNA from the indicated viral genes were measured by RT-qPCR. All samples were normalized to 18S and R443I-infected levels set to 1. (TIF) [file ppat.1008269.s003.tif]

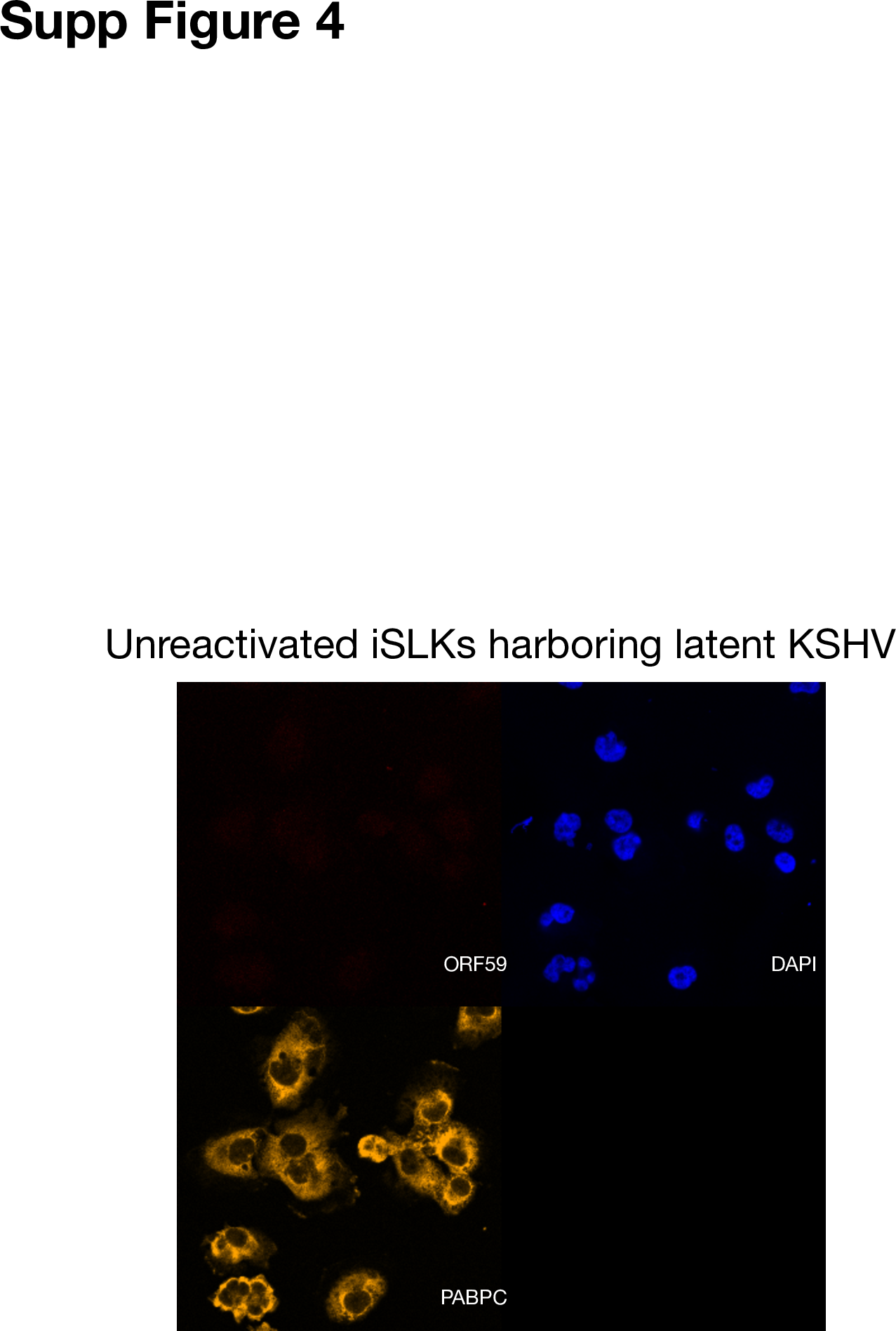

Supplement: S4 Fig — An immunofluorescence assay was performed on unreactivated (latent) KSHV-positive iSLK cells using antibodies against PABPC and the viral lytic protein ORF59. DNA was stained with DAPI. (TIF) [file ppat.1008269.s004.tif]
